# Crizotinib and its enantiomer suppress ferroptosis by decreasing PE-O-PUFA content

**DOI:** 10.1038/s41420-024-02127-8

**Published:** 2024-08-12

**Authors:** Si-Yu Cen, Fang Lin, Xuan Li, Yanglin Hu, Jin-Pin Liu, Zian Xue, Yun Gao, Yi-Ping Sun, Sanyong Zhu, Yongjun Dang, Yahui Zhao, Hai-Xin Yuan

**Affiliations:** 1 The Fifth People’s Hospital of Shanghai, Molecular and Cell Biology Laboratory, Institutes of Biomedical Sciences, Fudan University, Shanghai, 200032 China; 2 College of Pharmacy & Department of Cancer Center, the Second Affiliated Hospital, Chongqing Medical University, Chongqing, 400016 China; 3 Basic Medicine Research and Innovation Center for Novel Target and Therapeutic Intervention (Ministry of Education), Chongqing Medical University, Chongqing, 400016 China; 4 Department of Nephrology,, Wuhan No.1 hospital, Wuhan, 430022 China; 5 Department of Medicinal Chemistry, School of Pharmacy, Fudan University, Shanghai, 200120 China

**Keywords:** Cell death, Lipidomics

## Abstract

Ferroptosis is a specific form of cell death characterized by excessive accumulation of cellular lipid peroxides. Ferroptosis is closely associated with various diseases, inhibition of which may help alleviate multi-organ injury caused by ischemia-reperfusion and enhance the anti-tumor effect by promoting the immunity of T cells. However, clinical approved drugs targeting ferroptosis process remain rare. In this study, we unexpectedly found that (R)-crizotinib, the first-generation ALK inhibitor, has potent inhibitory activity against ferroptosis across various cell lines. Moreover, its chiral molecule (S)-crizotinib, which was considered to share no common targets with (R)-crizotinib, also suppresses ferroptosis with an efficacy similar to that of (R)-crizotinib. We further demonstrated that both crizotinib enantiomers inhibit ferroptosis independently of their known targets, but through a common mechanism involving the targeting of AGPAT3-mediated synthesis of ether-linked polyunsaturated fatty acids (PE-O-PUFA), which are known to promote lipid-ROS generation and ferroptosis. In line with their activity in cell lines, (R)-crizotinib and (S)-crizotinib effectively mitigate renal ischemia-reperfusion injury in mice. Furthermore, the two compounds also inhibit lipid-ROS accumulation in CD8^+^ T cells in draining lymph nodes of B16-F10 subcutaneous xenograft mice, thereby promoting anti-tumor effects. Collectively, our study firstly reports a common activity shared by (R)-crizotinib and (S)-crizotinib in ferroptosis regulation. As a clinically approved drug, (R)-crizotinib has well-established pharmacokinetics and safety, which makes it a promising candidate for repurposing. Given the current lack of FDA-approved ferroptosis inhibitors, our findings suggest therapeutically repurposing (R)-crizotinib as well as its enantiomer (S)-crizotinib for treating ferroptosis-related diseases.

## Introduction

Ferroptosis is a type of non-apoptotic cell death characterized by the accumulation of iron-dependent lipid peroxides [1]. Several biological processes, including System Xc^−^/GSH/GPX4 axis, iron metabolism and lipid metabolism are suggested to be involved in ferroptosis regulation [2]. A number of studies has demonstrated targeting ferroptosis improved various pathological conditions, such as NASH [3], neurodegenerative diseases [4] and multi-organ ischemia-reperfusion injury [5–7]. Furthermore, CD36 triggers uptake of fatty acids in CD8^+^ T cells resulting in ferroptosis, which consequently being unable to exert their anti-tumor effect [8, 9]. It has also been demonstrated that ferroptosis inhibitor sustains T cell function in anti-tumor therapy [8, 10]. However, clinically approved ferroptosis inhibitors remain scarce, which warrants the development of efficient and safe inhibitors of ferroptosis to meet clinical demand.

Crizotinib (also known as Xalkori, (R)-crizotinib, (R)-CRZ; PubChem CID: 11626560) is an ATP-competitive ALK inhibitor [11]. It has been approved by the U.S. FDA and in clinical practice as a targeted therapy for ALK-positive non-small cell lung cancer (NSCLC) [12]. The drug has demonstrated safety in years of clinical use, which benefits the majority of ALK-positive NSCLC patients, with an objective response rate of 60% and a progression-free survival of approximately 10 months [12]. Moreover, (R)-CRZ has been reported to exhibit inhibitory activity against some other receptor tyrosine kinases RTKs including c-Met, ROS1, and AXL [13–15].

(S)-crizotinib ((S)-CRZ, PubChem CID: 56671814) is the chiral form of (R)-CRZ. It is known to selectively targetable inhibiting MTH1 by suppressing the hydrolysis of cytotoxic oxidized nucleotides in DNA, leading to tumor cell death [16, 17]. A previous study has investigated targeting specificity between (R)-CRZ and (S)-CRZ. Notably, the two compounds exhibited significant differences in protein targeting, as (R)-CRZ has high targeting specificity for RTKs, while (S)-CRZ hardly binds to RTKs [18]. No common targets were found for (R)-CRZ and (S)-CRZ so far, despite there being only a single chiral difference between the two compounds.

In this study, we identified (R)-CRZ as a novel ferroptosis inhibitor through high-throughput compound screening. Interestingly, we observed that both (R)-CRZ and (S)-CRZ have inhibitory activity towards ferroptosis with similar efficacy, which is independent of their known targets. We further demonstrated that (R)-CRZ and (S)-CRZ may inhibit ferroptosis by decreasing AGPAT3-mediated PE-O-PUFA generation, a series of phosphatidylethanolamines containing polyunsaturated fatty acids on their side chains. (R)-CRZ and (S)-CRZ could ameliorate kidney ischemia-reperfusion injury in a mouse model [19], and they also eliminated lipid peroxides in CD8^+^ T cells to promote an anti-tumor effect, suggesting new therapeutic applications of (R)-CRZ and (S)-CRZ in treating ferroptosis-related diseases.

## Results

### (R)-CRZ suppresses ferroptosis in various cell lines independently of its known targets

**Fig. 1:**
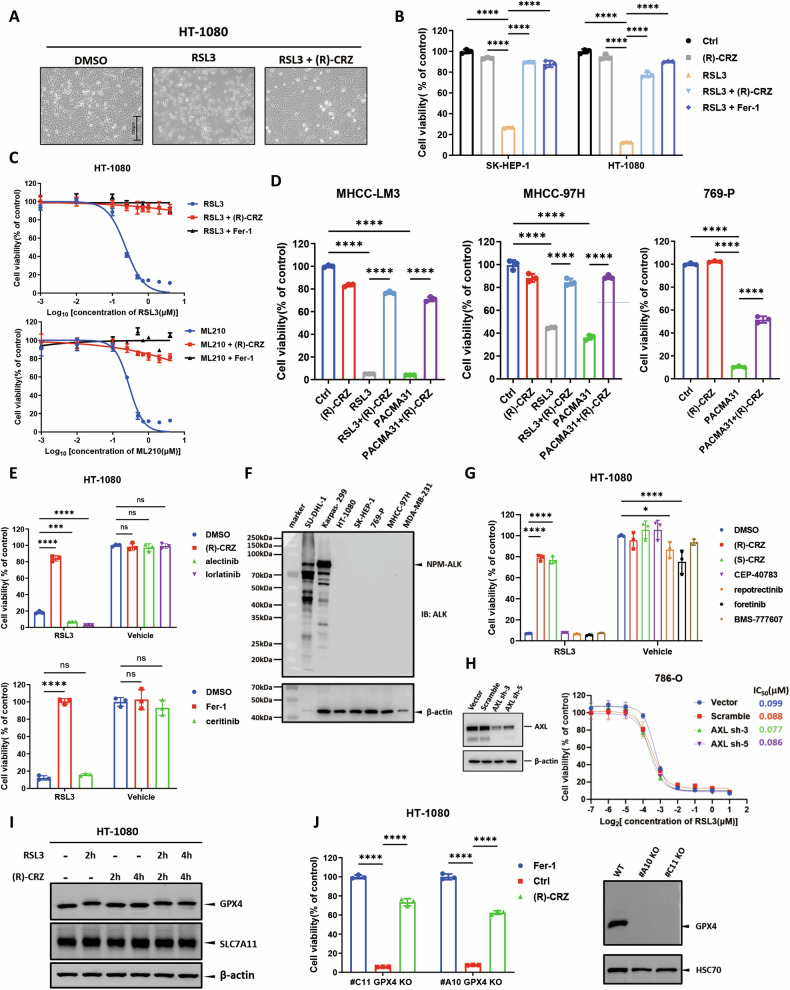
Crizotinib suppresses ferroptosis in various cell lines independently of ALK expression. **A** (R)-CRZ protects HT-1080 cells from RSL3-induced ferroptosis. HT-1080 cells were treated with RSL3 (0.25 μM) with or without (R)-CRZ (1 μM) added. Cell morphology was recorded at 24 h after drug treatment. **B** (R)-CRZ (1 μM) inhibits RSL3 (0.25 μM)-induced ferroptosis in both SK-HEP-1 and HT-1080 cell lines. Fer-1 (1 μM) was used as a positive control. Cell viability was measured after 10 h of treatment. **C** (R)-CRZ inhibits ferroptosis induced by RSL3 or ML210. (R)-CRZ (1 μM) and Fer-1 (1 μM) were used in the experiments. **D** (R)-CRZ (1 μM) inhibits ferroptosis induced by PACMA31 (1 μM) or RSL3 (1 μM) in multiple cell lines. Cell viability was assayed after 10 h of treatment. **E** Other ALK inhibitors do not inhibit ferroptosis. (R)-CRZ (3 μM), lorlatinib (3 μM), alectinib (3 μM), Fer-1 (1 μM) and ceritinib (1 μM) were used in the experiments. Cell viability was measured after 8 h of treatment. **F** Cell lines rescued from ferroptosis by (R)-CRZ did not show ALK expression as validated by Western blot. SU-DHL-1 and Karpas-299 cell lines were used as positive controls for ALK expression. **G** Some other RTK inhibitors do not affect ferroptosis. All tested compounds were used at a concentration of 3 μM in the experiment. **H** Knocking-down of *AXL* in 786-O cells do not affect cell sensitivity to ferroptosis. Cell viability was measured after 17 h of treatment. **I** (R)-CRZ (1 μM) do not affect GPX4 and SLC7A11 protein expression assayed by Western blot. **J** (R)-CRZ (3 μM) inhibits spontaneous ferroptosis in *GPX4* KO monoclonal HT-1080 cell lines. Cell viability was measured after 12 h of treatments. Data in all quantitative statistical charts of Fig. 1 are presented as the mean ± S.D., n = 3 independent repeats.

To identify novel ferroptosis inhibitors, we previously performed high-throughput compound screening using a library of more than 4000 bioactive compounds, which consists of FDA-approved drugs and natural compounds [2]. It is somewhat surprising that (R)-CRZ, a first-generation ALK inhibitor, showed potent inhibitory activity against RSL3-induced ferroptosis (Fig. 1A). Similar effect was confirmed in SK-HEP-1 cells, a hepatic adenocarcinoma cell line (Fig. 1B). Since RSL3 induces ferroptosis by covalently binding and suppressing GPX4 [20], two other GPX4-binding inhibitors, ML210 [21] and PACMA31 [22], were used to verify the effect of (R)-CRZ. Consistently, (R)-CRZ protected cells from ML210- or PACMA31- induced ferroptosis in multiple cell lines, including HT-1080, MHCC-97H (HCC cell line), MHCC-LM3 (HCC cell line) and 769-P (renal adenocarcinoma cell line) (Fig. 1C, D, Supplemental Fig. 1A). (R)-CRZ could also protect cell lines derived from different tissues, including breast cancer, prostate cancer and some non-tumor cell lines (Supplemental Fig. 1B), suggesting a general effect of (R)-CRZ in suppressing ferroptosis.

As a well-known drug targeting RTKs, we asked if (R)-CRZ suppresses ferroptosis through its known targets such as ALK, AXL, c-Met and ROS1 [13]. We first tested ceritinib, alectinib (second-generation ALK inhibitors) and lorlatinib (a third-generation ALK inhibitor) on ferroptosis, and found none of them inhibited ferroptosis (Fig. 1E). In addition, those cell lines that were protected by (R)-CRZ from ferroptosis do not have ALK expression (Fig. 1F), indicating that ALK is not involved in ferroptosis inhibition by (R)-CRZ. Next, we tested the effect of CEP-40783 (an inhibitor for c-Met and AXL), repotrectinib (a ROS1 inhibitor), foretinib (a c-Met kinase inhibitor) and BMS-777607 (an inhibitor for c-Met, AXL, RON and TYRO3) on ferroptosis. None of these compounds exhibited an inhibitory effect toward ferroptosis either (Fig. 1G). Consistently, knockdown of *AXL* in 786-O cells did not affect cell sensitivity to RSL3-induced ferroptosis (Fig. 1H). Taken together, these observations suggest a novel activity of (R)-CRZ in suppressing ferroptosis that is independent of its known targets.

We also examined whether (R)-CRZ’s inhibitory activity is dependent on GPX4, the key enzyme that eliminates cellular lipid peroxides. The protein levels of GPX4 and SLC7A11 were not affected by (R)-CRZ treatment (Fig. 1I). Furthermore, ferroptosis caused by *GPX4* knockout was rescued by either Fer-1 or (R)-CRZ (Fig. 1J), indicating (R)-CRZ functions in a GPX4 independent manner.

### Both (R)-CRZ and (S)-CRZ are specific inhibitors of ferroptotic cell death

**Fig. 2:**
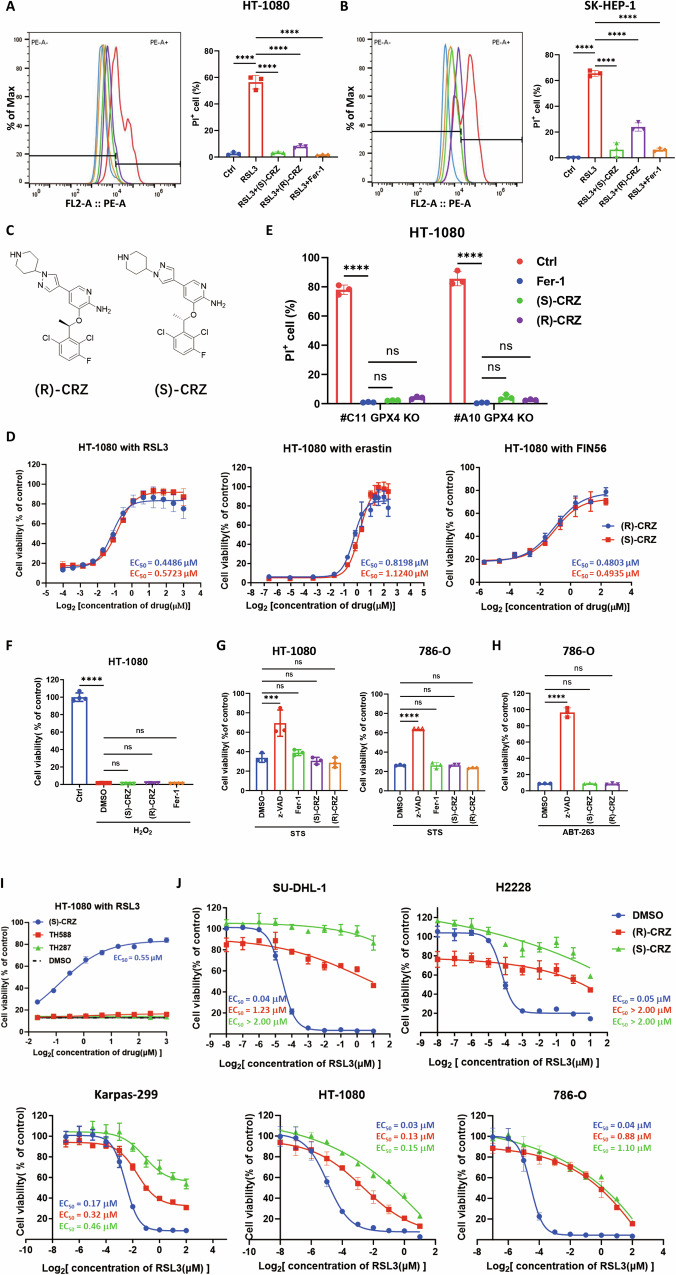
Both (R)-CRZ and (S)-CRZ are specific inhibitors of ferroptotic cell death. **A** (R)-CRZ and (S)-CRZ inhibited RSL3-induced ferroptosis in HT-1080 cells. Fer-1 (1 μM), (R)-CRZ (1 μM) and (S)-CRZ (1 μM) were used in the experiment. Cell death was detected by PI staining and FACS analysis at 8 h after treatment. **B** (R)-CRZ and (S)-CRZ inhibited RSL3-induced ferroptosis in SK-HEP-1 cells. The experiment was performed with the same conditions and analysis method used in **A**. **C** Chemical structures of (R)-CRZ and (S)-CRZ. **D** (R)-CRZ and (S)-CRZ have similar inhibitory effect on ferroptosis. EC_50_ of (R)-CRZ and (S)-CRZ against ferroptosis induced by RSL3, erastin or FIN56 in HT-1080 cells are indicated. For RSL3 treatment, 1 μM of drug was used and cell viability was measured at 10 h after treatment. A concentration of 10 μM of erastin was used and cell viability was measured11 h after treatment. For FIN56, 5 μM of drug was used to induce ferroptosis and cell viability was measured at 17 h after treatment. **E** (R)-CRZ (3 μM) and (S)-CRZ (3 μM) inhibited spontaneous ferroptosis in *GPX4* KO monoclonal HT-1080 cell lines. Cell death was measured after 10 h of treatments. **F** (R)-CRZ (3 μM) and (S)-CRZ (3 μM) do not protect HT-1080 cells from H_2_O_2_ (3 mM)-induced necrosis. Cell viability was measured at 8 h after treatment. Data are presented as the mean ± S.D., n = 4 independent repeats. **G** (R)-CRZ (3 μM) and (S)-CRZ (3 μM) did not protect HT-1080 cells from STS-induced apoptosis. z-VAD, a known apoptosis inhibitor, used as a positive control at 50 μM to inhibit apoptosis. Cell viability of HT-1080 was tested at 16 h after drug treatment. STS (12 μM) was used in 786-O cell line for 12 h to induce apoptosis. **H** (R)-CRZ (3 μM) and (S)-CRZ (3 μM) did not protect 786-O cells from ABT-263 (20 μM)-induced apoptosis. 50 μM of z-VAD was used as a positive control at to inhibit apoptosis. Cell viability was tested at 8 h after drug treatment. **I** Other MTH1 inhibitors (TH588 and TH287) do not suppress ferroptosis. Cell viability was tested at 10 h after drug treatment. EC_50_ of TH588 and TH287 cannot be calculated. **J** (R)-CRZ and (S)-CRZ inhibit ferroptosis in both ALK^+^ (Karpas-299, SU-DHL-1 and H2228) and ALK^-^ (HT-1080 and 786-O) cell lines. (R)-CRZ (3 μM) and (S)-CRZ (3 μM) were used to treat cells with different concentration of RSL3. Because the measurements in some groups did not reach the plateau, those EC_50_ calculations represent approximate values. Data in **A**, **B**, **D**, **E**, **G**–**J** are presented as the mean ± S.D., n = 3 independent repeats.

We noticed in previous experiment that (S)-CRZ also inhibit ferroptosis as (R)-CRZ did (Fig. 1G). (S)-CRZ also exhibited similar ferroptosis inhibitory effect among various cell lines (Fig. 2A–C, Supplemental Fig. 1A, B). As this might be the first time that (R)-CRZ and (S)-CRZ exhibit similar activity, we confirmed this effect on ferroptosis induced by RSL3, erastin or FIN56 (Fig. 2D). Notably, (S)-CRZ and (R)-CRZ exhibited similar EC_50_ in ferroptosis inhibition. Similar to (R)-CRZ, (S)-CRZ also inhibited spontaneous ferroptosis in *GPX4* knockout cells (Fig. 2E, Supplemental Fig. 1C). Interestingly, this inhibitory activity towards cell death seems to be specific to ferroptosis, as both forms of crizotinib did not rescue cells from H_2_O_2_-induced necrosis (Fig. 2F), staurosporine (STS)-induced apoptosis (Fig. 2G) or ABT-263-induced intrinsic apoptosis (Fig. 2H). MTH1, a nucleotide pool sanitizing enzyme, is the only reported target of (S)-CRZ [18]. Our experiment showed that MTH1 inhibitors TH588 and TH287 did not rescue cells from ferroptosis (Fig. 2I), suggesting MTH1 is not responsible for ferroptosis inhibition by (S)-CRZ. Thus, both (S)-CRZ and (R)-CRZ exhibit similar efficacy in suppressing ferroptosis possibly through novel mechanisms.

It has been reported that (S)-CRZ does not target RTKs like (R)-CRZ. We compared the effect of (S)-CRZ and (R)-CRZ on ferroptosis in several ALK^+^ (Karpas-299, SU-DHL-1 and H2228) or ALK^−^ (HT-1080 and 786-O) cancer cell lines. In line with previous results, both enantiomers of crizotinib rescued all five cell lines from RSL3-induced ferroptosis with similar efficacy (Fig. 2J). It is notable that (R)-CRZ treatment significantly reduced the viability of ALK^+^ cell lines, which is consistent with its potent activity as an ALK inhibitor (Fig. 2J). These data indicate the dual role of (R)-CRZ in inhibiting ALK-dependent proliferation as well as ferroptosis in ALK^+^ cells.

Collectively, these observations indicate that the inhibitory activity towards ferroptosis is a novel function of crizotinib enantiomers, which is independent of their known targets. Although comparison profiles of (R)-CRZ and (S)-CRZ did not reveal any common targets bound to both enantiomers [18], the fact that they have comparable EC_50_ for ferroptosis inhibition suggested that these two compounds may have a common target which played a role in ferroptosis regulation.

### Both (S)-CRZ and (R)-CRZ inhibit ferroptosis not through direct antioxidant activity or by affecting iron level

**Fig. 3:**
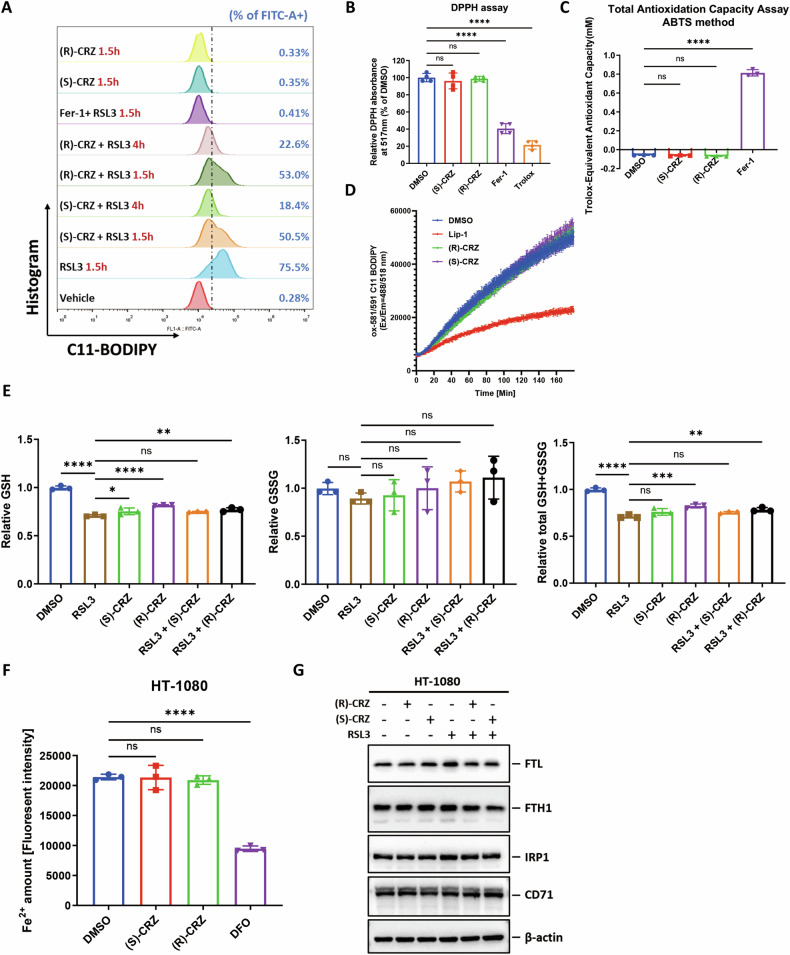
(R)-CRZ and (S)-CRZ inhibit ferroptosis not through direct antioxidant activity or by affecting iron level. **A** (S)-CRZ (1 μM) and (R)-CRZ (1 μM) inhibit RSL3 (0.25 μM)-induced lipid-ROS accumulation in 786-O cells. Cellular lipid-ROS was measured by C11-BODIPY 581/591 (1 µM) staining coupled with flow cytometry analysis. Fer-1 (1 μM) was used as a positive control for lipid-ROS elimination. **B** Both (S)-CRZ (50 μM) and (R)-CRZ (50 μM) have no antioxidant capacity in vitro by 2,2-Diphenyl-1-picrylhydrazyl (DPPH) assay. The antioxidant capacity of indicated compounds was measured by scavenging DPPH. Trolox (50 μM) and Fer-1 (50 μM) were used as positive controls. **C** Both (S)-CRZ and (R)-CRZ have no antioxidant capacity in vitro by ABTS test. Each compound in this ABTS test was used at 1 mM. **D** Both (S)-CRZ and (R)-CRZ do not suppress the autoxidation of liposomes. Autoxidation of C11-BODIPY 581/591 (1 µM) and liposomes of egg phosphatidyl-choline lipids (1 mM) suspended in PBS (pH 7.4) was initiated by adding 1 mM AAPH. Indicated compounds (1 µM) were added to the reaction individually, and fluorescence signal of C11-BODIPY 581/591 was monitored. **E** (S)-CRZ and (R)-CRZ do not affect GSH and GSSG levels substantially. HT-1080 cells were treated with RSL3 for 2.5 h to induce ferroptosis. (S)-CRZ (3 μM) and (R)-CRZ (3 μM) were used in these experiments. GSH, GSSG and total (GSH + GSSG) level were measured as described in the Methods. **F** (S)-CRZ (3 μM) and (R)-CRZ (3 μM) treatment do not change cellular iron levels. Iron levels were measured by a Fe^2+^ indicator FerroOrange (1 μM). DFO (100 μM) was used as a positive control. **G** (S)-CRZ (3 μM) and (R)-CRZ (3 μM) do not affect the expression of genes involved in iron metabolism. Protein expression levels were validated by Western blot. Data in **B**–**F** are presented as the mean ± S.D., n = 3 independent repeats.

Next, we explored how (S)-CRZ and (R)-CRZ may regulate ferroptosis process. Lipid-ROS level was measured by using the fluorescent probe C11-BODIPY (581/591). Consistent with ferroptosis suppression, RSL3-induced lipid-ROS accumulation was inhibited by both crizotinib enantiomers (Fig. 3A). It is notable that compared to Fer-1 which almost completely eliminated lipid-ROS within 1.5 h, (S)-CRZ and (R)-CRZ reduced lipid-ROS level at a much lower rate (over 4 h), indicating crizotinib enantiomers might not function as free radical scavengers as Fer-1 that directly eliminates lipid-ROS. We verified this point by analyzing the antioxidant capacity of both forms of crizotinib using the DPPH ((2,2-Di(4-tert-octylphenyl)-1-picrylhydrazyl)) assay and the ABTS (Trolox equivalent antioxidant capacity, TEAC assay) test. Indeed, both (R)-CRZ and (S)-CRZ did not exhibit antioxidant capability like Fer-1 did (Fig. 3B, C).

To better demonstrate the lipid substrates oxidation process in ferroptosis, a liposome experiment was performed using autoxidized egg phosphatidylcholine as a substrate. Lip-1, as a positive control, significantly suppressed the oxidation of egg phosphatidylcholine, whereas neither (R)-CRZ nor (S)-CRZ reduced the generation of lipid peroxidation (Fig. 3D).

We also examined whether crizotinib’s inhibitory activity is dependent on the level of GSH. RSL3 caused a mild decrease in GSH and total (GSH + GSSG) level, whereas (R)-CRZ and (S)-CRZ treatment only had slight effect on the amount of GSH and total (GSH + GSSG) compared to RSL3 treatment (Fig. 3E), indicating that crizotinib enantiomors inhibit ferroptosis not through affecting GSH metabolism.

We also measured cellular labile ferrous ion level, which is essential for ferroptosis occurence [23]. Compared to iron chelator deferoxamine (DFO), neither (S)-CRZ nor (R)-CRZ affect Fe^2+^ level (Fig. 3F). Furthermore, crizotinib enantiomers did not affect the expression of iron metabolism related genes including FTL, FTH1, IRP1 and CD71 (Fig. 3G). Collectively, these observations indicated that (S)-CRZ and (R)-CRZ do not inhibit ferroptosis through antioxidant activity or affecting iron metabolism.

### (S)-CRZ and (R)-CRZ inhibit ferroptosis by reducing PE-O-PUFA

**Fig. 4:**
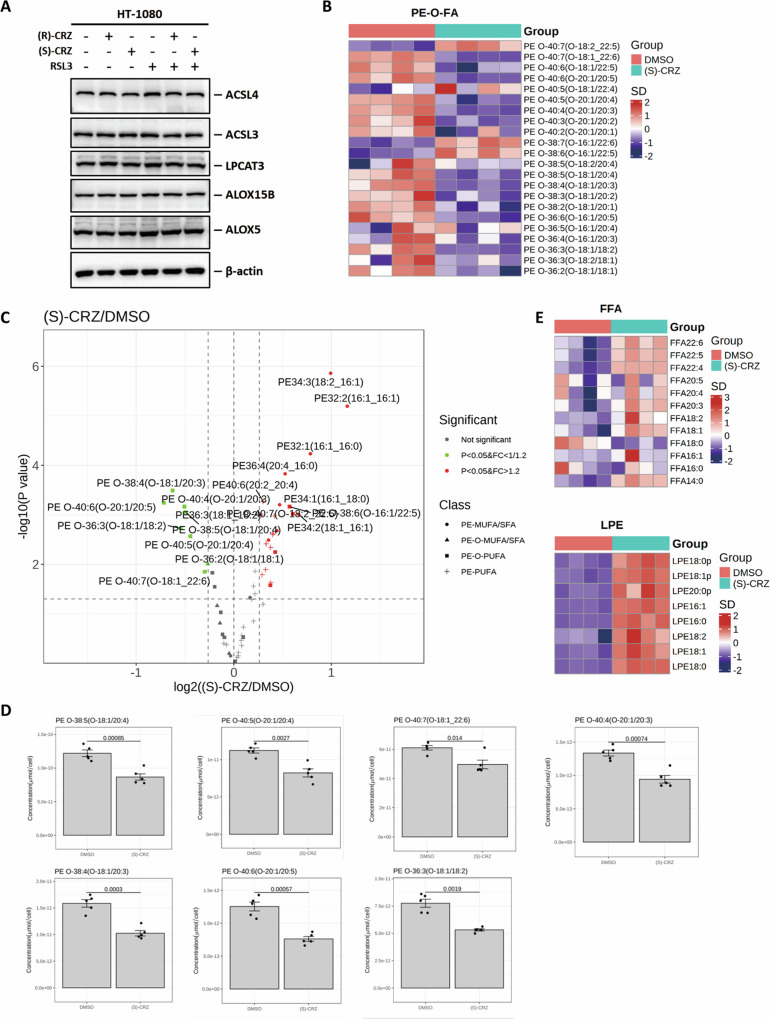

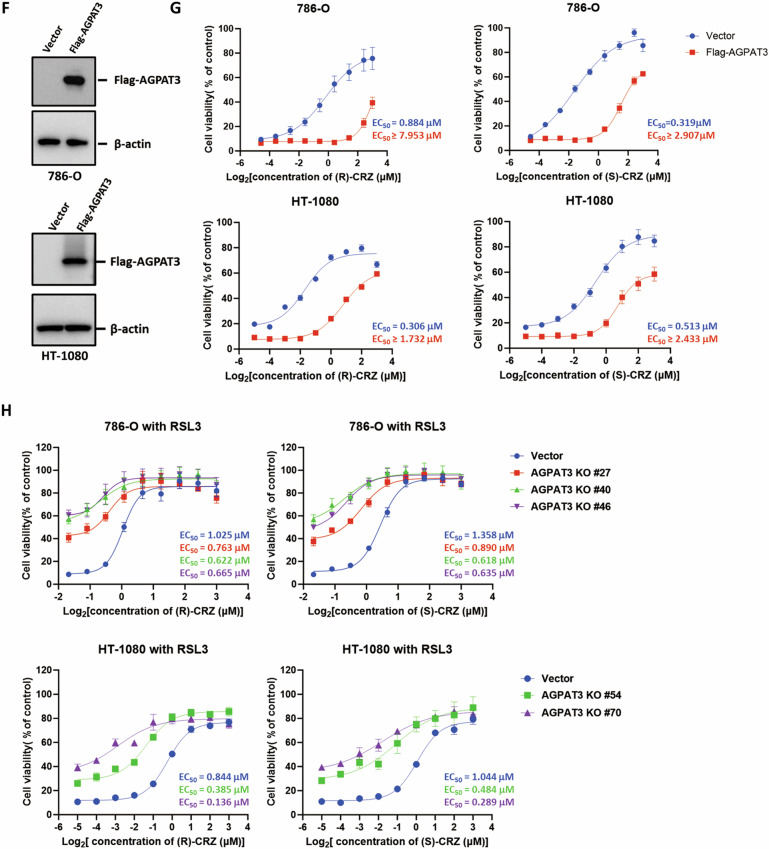
(S)-CRZ inhibits ferroptosis by decreasing PE-O-PUFA. **A** (S)-CRZ (3 μM) and (R)-CRZ (3 μM) do not affect the expression of ferroptosis-related lipid-metabolism genes validated by Western blot. (**B–D**) (S)-CRZ inhibits ferroptosis by decreasing PE-O-PUFA. This test consisted of unpaired independent samples of DMSO and (S)-CRZ (3 µM) groups (n = 4), and the hypothesis test used was a non-parametric test Mann-Whitney *U* test. Heatmap analysis showed decrease of ether-linked-PE components in lipidomic profile of HT-1080 cells upon (S)-CRZ treatment compared to DMSO group (**B**). Volcano plot showed differential PE components in lipidomic profile of HT-1080 cells line upon (S)-CRZ treatment compared to DMSO group (**C**). Amount of indicated PE-O-PUFA in HT-1080 cells were shown upon (S)-CRZ or DMSO treatment (**D**). **E** Heatmap analysis showed increase of free polyunsaturated fatty acid and LPE components in lipidomic profile of HT-1080 cells upon (S)-CRZ treatment compared to DMSO group. **F** Validation of Flag-AGPAT3 expression in 786-O and HT-1080 cells by Western blot. **G** AGPAT3 overexpression reduced potency of (S)-CRZ and (R)-CRZ towards RSL3 induced ferroptosis. **H** Knockout of *AGPAT3* increased potency of (S)-CRZ and (R)-CRZ towards RSL3-induced ferroptosis. Cell viabilities were detected at appropriate time. Data in **G**, **H** are presented as the mean ± S.D., n = 3 independent repeats.

Since crizotinib enantiomers reduced lipid-ROS levels (Fig. 3A), it is conceivable that they may affect the process of lipid-ROS generation. (S)-CRZ and (R)-CRZ did not change the expression of genes in ferroptosis-related lipid metabolism (Fig. 4A). To determine whether (S)-CRZ and (R)-CRZ inhibit ferroptosis through affecting lipid contents, we performed a lipidomic analysis on HT-1080 cells treated with DMSO or (S)-CRZ. Interestingly, the class of ether-linked polyunsaturated fatty acid phosphatidylethanolamines (PE-O-PUFAs) was significantly downregulated upon (S)-CRZ treatment, especially those containing C20:4 (arachidonic acid), C20:5, C18:2, C20:3 and C22:6 (DHA, docosahexaenoic acid) as side chain (Fig. 4B, C). The concentration of each PE-O-PUFA was shown in the histogram (Fig. 4D). Meanwhile, free polyunsaturated fatty acid and LPE, two substrates for PE-O-PUFA generation [24], were accumulated in cells treated by (S)-CRZ (Fig. 4E).

PUFA chain is known to be critical for lipid-ROS generation and ferroptosis sensitization [25], including C20:4 [26] and C22:6 [27]. Notably, it has been reported that selective incorporation of arachidonic acid or docosahexaenoic acid into lysophosphatidic acids, a process catalyzed by AGPAT3 [24, 28], facilitates lipid-ROS generation and promotes ferroptosis [25]. We thus hypothesize (S)-CRZ and (R)-CRZ might inhibit ferroptosis by targeting AGPAT3-mediated generation of PE-O-PUFAs. To verify this, we overexpressed AGPAT3 in 786-O and HT-1080 cells and found that AGPAT3 overexpression increased sensitization to RSL3-induced ferroptosis (Supplemental Fig. 2B) and significantly reduced the potency of both enantiomers of crizotinib in suppressing RSL3-induced ferroptosis as demonstrated by the increase in EC_50_ by over 5–10 folds (Fig. 4F, G). In line with a previous study [25], *AGPAT3* knockout increased resistance to ferroptosis (Supplemental Fig. 2). Importantly, *AGPAT3* knockout increased the potency of crizotinib enantiomers, as demonstrated by a decrease in EC_50_ by 2–4 folds in the two cell lines (Fig. 4H). It is noteworthy that crizotinib enantiomers still exhibited a protective effect at high concentrations in *AGAPT3* knockout cells (Fig. 4H), suggesting the presence of additional mechanisms by which crizotinib enantiomers suppress ferroptosis.

### Both (S)-CRZ and (R)-CRZ ameliorate renal ischemia-reperfusion injury in a mouse model

**Fig. 5:**
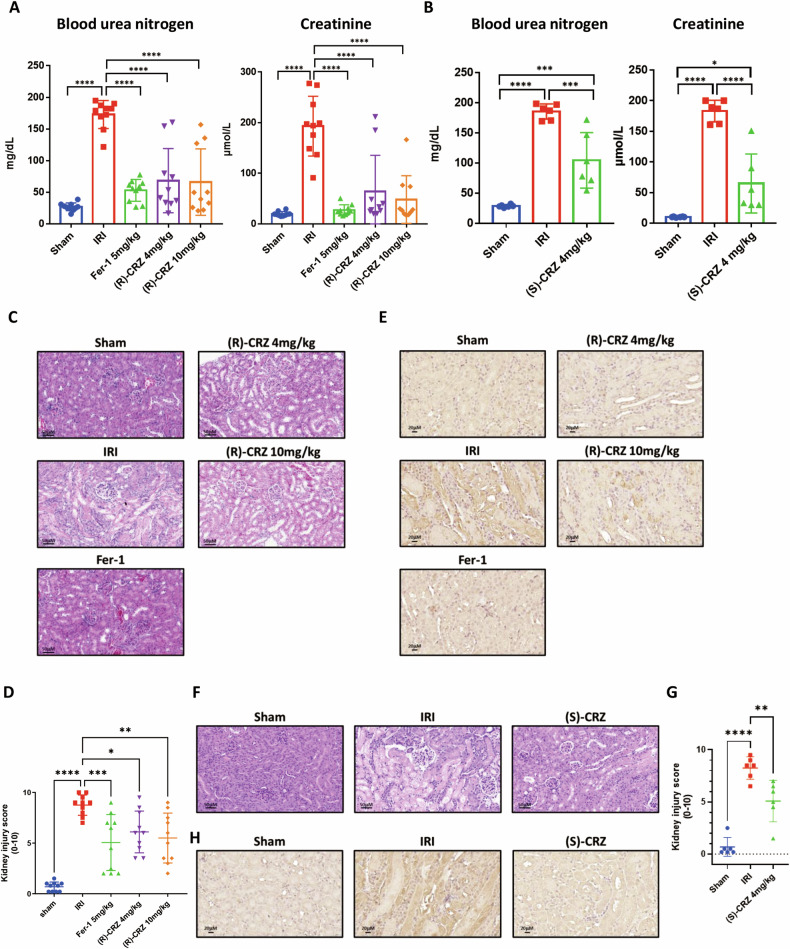
(R)-CRZ and (S)-CRZ ameliorate renal ischemia-reperfusion injury (IRI) caused by ferroptosis in a mouse model. **A**, **B** (R)-CRZ **A** and (S)-CRZ **B** ameliorate renal ischemia reperfusion injury (IRI) indicated by reduced serum urea nitrogen and creatinine levels. **C**, **D** Fer-1 and (R)-CRZ ameliorate kidney damage caused by IRI. **E** (R)-CRZ decreases the 4-HNE signal in the mouse kidney with ischemia-reperfusion injury. **F**, **G** (S)-CRZ ameliorate kidney damage caused by IRI. **H** (S)-CRZ decreases the 4-HNE signal in the mouse kidney with ischemia-reperfusion injury. Representative Hematoxylin and eosin (H & E) staining pictures showed pathological morphology of kidney tissue (**C**, **F**). Quantification of renal damage from mice of indicated groups (**D**, **G**). Representative immunohistochemistry (IHC) staining pictures of 4-HNE in kidney sections from control and compounds pretreated mice were shown (**E**, **H**).

To demonstrate the therapeutic effect of crizotinib enantiomers as ferroptosis inhibitors, we tested their effect on a mouse model of renal ischemia-reperfusion injury, a common pathological process involves ferroptosis [19, 29]. Increases of serum creatinine and blood urea nitrogen levels were detected 24 h after kidney ischemia-reperfusion in operating group, which were significantly mitigated by treatment with (S)-CRZ or (R)-CRZ (Fig. 5A, B), indicating that both (R)-CRZ and (S)-CRZ alleviated ischemia-reperfusion-induced kidney damage. Consistently, histological analysis revealed significant improvement in kidney injury in the treatment group compared to the control group (Fig. 5C, D, F, G). Immunohistochemical analysis showed that ischemia-reperfusion injury resulted in an increased level of 4-hydroxynonenal (4-HNE), a molecular marker of lipid peroxidation, which was significantly reduced by treatment with both crizotinib enantiomers and Fer-1 (Fig. 5E, H). These observations demonstrate that enantiomers of crizotinib function in vivo to ameliorate renal ischemia-reperfusion injury.

### Both (S)-CRZ and (R)-CRZ reduce lipid peroxides in CD8^+^ T cells to enhance the anti-tumor effect in the B16-F10 subcutaneous xenograft mouse model

**Fig. 6:**
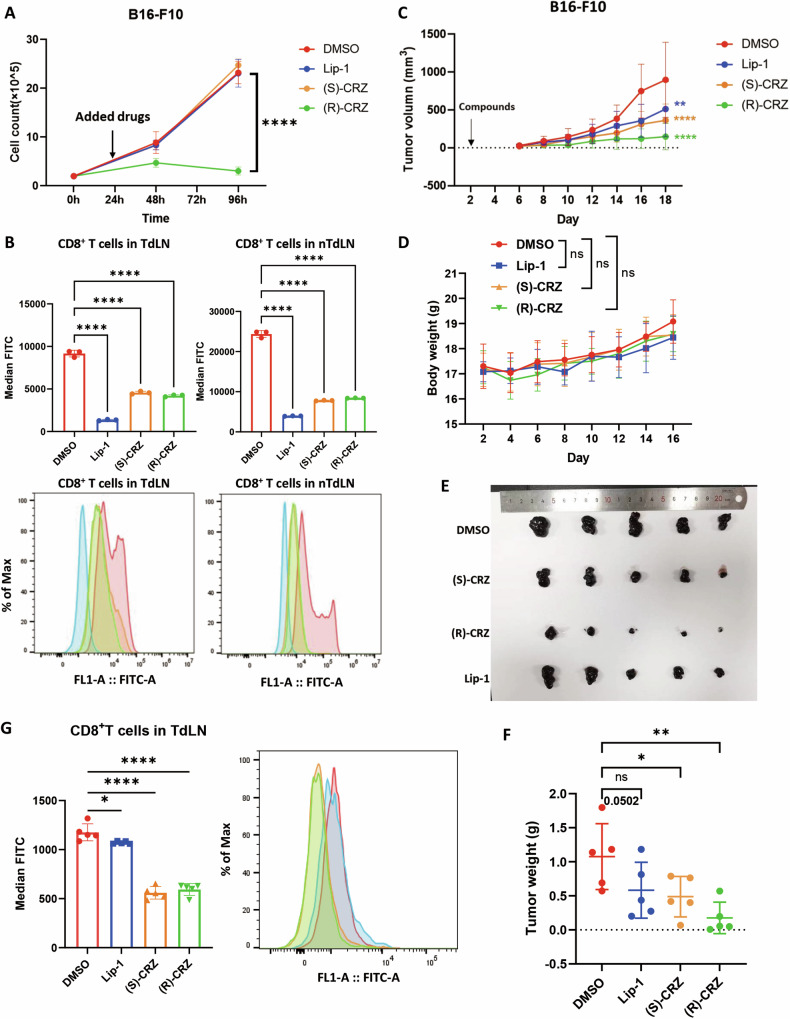
(S)-CRZ and (R)-CRZ reduce lipid peroxides in CD8^+^ T cells to promote anti-tumor effect. **A** (S)-CRZ (1 μM) or Lip-1 (1 μM) do not suppress the proliferation of B16-F10 cells, whereas (R)-CRZ (1 μM) exhibits cytotoxicity to B16-F10 cells. Statistical significance was assessed by comparing each group with the DMSO group at 96 h, distinguished by color. **B** Lip-1 (2 µM), (S)-CRZ (2 µM) and (R)-CRZ (2 µM) treatment reduce lipid-ROS in CD8^+^ T cells isolated from TdLNs and nTdLNs of B16-F10 tumor subcutaneous bearing mice. Lymph cells from lymph nodes separated from tumor-bearing mice were treated with indicated compounds for 3 h followed by flow cytometry analysis. Lipid-ROS was detected by fluorescence of C11-BODIPY 581/591 at the FITC channel. Data in **A**, **B** were presented as the mean ± S.D., n = 3 independent repeats. (**C**–**F**) Intraperitoneal injection of (S)-CRZ and (R)-CRZ suppress growth of B16-F10 xenograft tumors. B16-F10 tumor growth with indicated treatment (DMSO; 5 mg/kg (S)-CRZ; 5 mg/kg (R)-CRZ; 10 mg/kg Lip-1) (**C**). Body weight of mouse with indicated treatment (**D**). Photograph of B16-F10 tumors after excision from each group (**E**). Tumor weight of different treatment groups after excision (**F**). **G** (S)-CRZ (5 mg/kg) and (R)-CRZ (5 mg/kg) treatment significantly reduce lipid-ROS in CD8^+^ T cells from TdLNs of B16-F10 tumor subcutaneous bearing mice. CD8^+^ T cells were isolated from TdLNs of tumor-bearing mice with intraperitoneal drug treatment for 16 d. Lipid-ROS was detected by fluorescence of C11-BODIPY 581/591 in the FITC channel. Data in **C**–**G** are presented as the mean ± S.D., n = 5 independent repeats.

It has been reported that CD8^+^ T cells accumulate high levels of lipid peroxides, which impairs their anti-tumor capabilities. Ferroptosis inhibitors help clear lipid-ROS to maintain CD8^+^ T cells function [8, 9]. Given the novel activity of (S)-CRZ and (R)-CRZ in suppressing ferroptosis, we investigated whether these compounds could benefit anti-tumor immunity. Therefore, we evaluated the effects of (S)-CRZ and (R)-CRZ in a subcutaneous xenograft mouse model using B16-F10 cells, a mouse melanoma cell line. Lip-1 and (S)-CRZ did not inhibit the proliferation of B16-F10 cells under culture condition (Fig. 6A), while (R)-CRZ, as along with other RTK inhibitors, suppressed the proliferation of B16-F10 cells (Fig. 6A, Supplemental Fig. 3A), indicating the potent activity of (R)-CRZ as a RTK inhibitor in the B16-F10 cell line. CD8^+^ T cells were isolated from either tumor-drained lymph node (TdLN) or non-tumor-drained lymph node (nTdLN) of B16-F10 subcutaneous xenograft tumor mice and treated with compounds ex vivo. Consistent with observations in cultured cells, both (S)-CRZ and (R)-CRZ significantly decreased lipid-ROS accumulation in CD8^+^ T cells from both TdLN and nTdLN, similar to the effect of Lip-1 (Fig. 6B). In line with this, (S)-CRZ and (R)-CRZ also significantly suppressed tumor progression as Lip-1 did (Fig. 6C–F). Notably, Lip-1 and (S)-CRZ inhibited tumor growth to a similar extent whereas (R)-CRZ exhibited a stronger inhibitory effect on tumor growth, which supported the notion that (R)-CRZ may have dual roles in targeting RTKs in cancer cells and preventing ferroptosis in CD8^+^ T cells. Additionally, both (S)-CRZ and (R)-CRZ reduced lipid peroxides in CD8^+^ T cells from TdLN in vivo after prolonged drug treatment (16 days) (Fig. 6G). Furthermore, (S)-CRZ and (R)-CRZ were more potent than Lip-1 in suppressing lipid peroxides accumulation. In addition, both (S)-CRZ and (R)-CRZ also eliminated lipid-ROS in CD4^+^ T cells both under ex vivo or in vivo condition (Supplemental Fig. 3B, C). Taken together, these results demonstrate that both (S)-CRZ and (R)-CRZ reduce lipid peroxides in CD8^+^ T cells, potentially preserving CD8^+^ T cells function and promoting their anti-tumor effects.

## Discussion

In this study, we report an unexpected activity of (S)-CRZ and (R)-CRZ as potent ferroptosis inhibitors, and this new activity is independent of their known targets. Both (S)-CRZ and (R)-CRZ exhibit similar EC_50_ values for ferroptosis inhibition, indicating a common mechanism for the enantiomers in the regulation of ferroptosis. Furthermore, we verified the in vivo ferroptosis inhibitory effect of (S)-CRZ and (R)-CRZ in ameliorating renal ischemia-reperfusion injury as well as benefiting tumor immunotherapy. To our knowledge, this is the first time demonstrating a common pharmacology activity of both crizotinib enantiomers.

Evaluation of (R)-CRZ in ALK-positive cell lines confirms its dual role in inhibiting both cell proliferation and ferroptosis. In fact, numerous studies have reported that many compounds in clinical or pre-clinical state induce ferroptosis as part of their mechanism, such as sorafenib [30, 31], vorinostat [32], artesunate [33] and sulfasalazine [34]. Furthermore, several studies have proved utilization of vulnerability to ferroptosis in several cancers is a potential strategy [34–36]. Given the growing interest in exploiting ferroptosis susceptibility for cancer treatment, our study raises concern about potential diminished therapeutic efficacy when combining (R)-CRZ with ferroptosis inducers in cancer treatment.

Through lipidomic analysis, we demonstrated that (S)-CRZ downregulates phosphatidylethanolamine species containing ether-bonded polyunsaturated fatty acids. Consistently, modulation of AGPAT3-mediated generation of PE-O-PUFAs significantly altered the potency of both (S)-CRZ and (R)-CRZ. Previous reports have linked similar lipid compositions to ferroptosis sensitivity regulated by AGPAT3 [25]. However, an increase of cellular resistance to ferroptosis was observed under high dose of (S)-CRZ or (R)-CRZ treatment in *AGPAT3* knockout cells, which implies that AGPAT3 pathway is not the sole target of crizotinib enantiomers. Moreover, we cannot definitively conclude whether AGPAT3 is a direct target of crizotinib enantiomers. Indeed, there remains a possibility that (S)-CRZ and (R)-CRZ suppress lipid-ROS and ferroptosis by regulating pathways parallel to AGPAT3-mediated PE-O-PUFAs generation. Further studies are needed to elucidate the detailed mechanisms of (S)-CRZ and (R)-CRZ on PE-O-PUFA metabolism and ferroptosis inhibition.

Notably, the inhibitory effect of (S)-CRZ and (R)-CRZ on ferroptosis were validated in murine models. Ferroptosis has been reported to play a critical role in acute renal ischemia-reperfusion process [19, 29], which can be ameliorated by (S)-CRZ and (R)-CRZ (Fig. 5). Besides, the benefits of ferroptosis inhibitor on tumor immunotherapy have been documented. It has been demonstrated that clearing lipid-ROS stress in CD8^+^ T cell promotes its immunity [8, 9]. Another study also proved that reducing ROS levels increases the cytotoxicity of T cells and alleviates exhaustion [37]. In our xenograft melanoma tumor model, both enantiomers of crizotinib inhibited tumor growth over a 16-day drug administration period. While Lip-1 exhibited stronger inhibition of lipid peroxidation in CD8^+^ T cell ex vivo, both (R)-CRZ and (S)-CRZ significantly overperformed Lip-1 in in vivo assessments. Considering the inherent role of (R)-CRZ as an RTK inhibitor, this discovery expands its potential clinical applications by simultaneously inhibiting cancer cell proliferation through RTK inhibition and promoting anti-tumor immunity by alleviating ferroptosis-induced stress in CD8^+^ T cells.

Furthermore, we also observed the clearance of lipid-ROS in CD4^+^ T cells both ex vivo and in vivo (Supplemental Fig. 3). CD4^+^ T cells, including helper T cells and regulatory T cells, play vital roles in immune response against infection and disease, which interact and activate other cells in the immune system [38]. Although the relationship between ferroptosis of CD4^+^ T cells and anti-tumor effect is not clear so far, studies have reported that ferroptotic stress is harmful for CD4^+^ T cells’ function in defending against virus infection [39–41]. It could be speculated that clearance of lipid-ROS in CD4^+^ T cells may also benefit the anti-tumor immune response, which warrants in-depth investigations in future studies.

The evolving understanding of ferroptosis in various pathological processes underscores the need for effective and safe ferroptosis inhibitors for clinical use. However, there is still a lack of clinically approved ferroptosis inhibitors to date. Deferoxamine (DFO), an FDA-approved drug known for its iron-chelating properties and as a ferroptosis inhibitor, remains the only FDA-approved option. However, DFO has been reported to cause many side effects in clinical use, including impaired bone development [42], high-frequency sensorineural hearing loss [43, 44], changes in the retinal pigment epithelium (RPE), visual loss, and impaired night vision [45, 46]. Therefore, (R)-CRZ, with its established clinical history and FDA approved for safety, along with its enantiomer (S)-CRZ, offer promising alternatives.

In summary, our study highlights the ferroptosis inhibitory effect of (R)-CRZ and its enantiomer (S)-CRZ. Repurposing of (R)-CRZ and (S)-CRZ may provide valuable therapeutic strategies for ferroptosis-related diseases.

## Materials and methods

### Antibodies, plasmid, and chemicals

Antibodies used in this study for Western Blot were: GPX4 (Abcam, ab125066), β-actin (Proteintech, 66009-1-Ig), HSC70 (Proteintech, 10654-1-AP), SLC7A11 (CST, 12691), ALK (CST, 3791), AXL (CST, 8661), FTL (Abcam, ab69090), FTH1 (CST, 4393), IRP1 (CST,20272), CD71 (CST, 13113), ACSL4 (Proteintech, 66617-1-Ig), ACSL3 (Proteintech, 20710-1-AP), LPCAT3 (Proteintech, 67882-1), ALOX15B (Proteintech, 13073-1-AP) and ALOX5 (Proteintech, 66326-1-Ig).

Antibodies used in this study for Flow Cytometer against CD45 (BD Pharmingen, 557659), CD3e (BD Pharmingen, 551163), CD4 (BD Pharmingen, 553051), CD8 (BD Pharmingen, 553032) and Mouse CD16/CD32 (BD Pharmingen, 553141) were purchased commercially.

Other probes including Propidium iodide (PI, Sigma-Aldrich, P4170), Fixable Viability Stain 510 (BD Pharmingen, 564406) and C11-BODIPY 581/591 (Thermo Fisher Scientific, D3861) were purchased commercially.

Full-length cDNA of *AGPAT3*(NM_020132.5) was amplified by PCR and cloned into pLVX vector using standard protocols. All constructions were verified by DNA sequencing.

All chemicals used in this study were purchased commercially including erastin (Selleckchem, S7242), FIN56 (CSNpharm, CSN20583), RSL3 (1 S,3R-RSL3, Sigma-Aldrich, SML2234), Fer-1 (ferrostatin-1, Selleckchem, S7243), Lip-1 (liproxstatin-1, TargetMol, T2376), PACMA31 (Sigma-Aldrich, SML0838), (R)-crizotinib (PF-02341066, Selleckchem, S1068), (S)-crizotinib (Selleckchem, S7505), H_2_O_2_ (Sinopharm,10011208), staurosporine (MCE, HY-15141), DFO (MCE, HY-B0988), alectinib (Selleckchem, S2762), lorlatinib (PF-6463922, Selleckchem, S7536), ceritinib (TargetMol,T1791), CEP-40783(MCE, HY-100946), foretinib (TargetMol, T3113), repotrectinib (TargetMol, T4071), BMS-777607 (TargetMol, T2699), Navitoclax (ABT-263, Selleckchem, S1001), z-VAF-FMK(Selleckchem, S7023), DPPH (2,2-Di(4-tert-octylphenyl)-1-picrylhydrazyl, Sigma-Aldrich, 257621), L-α-phosphatidylcholine (egg, chicken) (Merck, 840051 P) and AAPH(2,2′-Azobis(2-methylpropionamidine) dihydrochloride, Merck, 440914).

### Cell culture, transfection, and stable cell lines generation

HT-1080, HEK293T, 786-O, 769-P, SK-HEP-1, PC-3, H9c2, ARPE-19, MDA-MB-231, SU-DHL-1, Karpas-299, H2228 and B16-F10 cells were purchased from the American Type Culture Collection (ATCC). MHCC-97H and MHCC-LM3 cells were purchased from the China National Collection of Authenticated Cell Cultures. All cell lines were verified without mycoplasma contamination. PC-3, SU-DHL-1, H2228, Karpas-299 cells were cultured in RPMI-1640 medium (Meilunbio, MA0215), ARPE-19 cells were cultured in DMEM: F-12 Medium (1:1, Meilunbio, MA0212, MA0229), and other cells were cultured in DMEM containing 4.5 g/L glucose and L-glutamine (Meilunbio, MA0212). HT-1080 *GPX4* KO cells were grown in DMEM supplemented with 1 μM of Fer-1. All media were supplemented with 10% fetal bovine serum (ExCell Bio) and 50 μg/mL of penicillin/streptomycin (Meilunbio), and all cell lines were grown at 37 °C with 5% CO_2_.

To generate *GPX4* or *AGPAT3* knockout monoclonal cells, virus was produced by cotransfection of HEK293T cells with psPAX, pMD2.G, and pLenti expression plasmids at a 3:1:4 w/w/w ratio. The medium containing secreted virus was harvested after 36 h and used to infect target cell lines with addition of polybrene. CRISPR guide RNA (sgRNA) sequences targeting *GPX4* and *AGPAT3* were designed using the online-available CRISPR design tool developed by the Zhang laboratory (http://crispr.mit.edu/). The sgRNA sequences were designed as follows:

AGPAT3-sgRNA: caccg TACCACGTCCAGCCGATGAG; and

GPX4-sgRNA: caccg CTTGGCGGAAAACTCGTGCA.

Seventy-five cells were seeded into 96-well plates to get single-clone cells. After about 2 weeks, single-clone cells were transferred to 24-well plates and gene expression in each clone was examined by Western blot and gene sequencing.

To generate 786-O stable cell lines with *AXL* knockdown, shRNA oligos targeting human *AXL* (NCBI Gene ID: 558) were custom-synthesized, annealed, and inserted into the pLKO.1 plasmid. Lentiviruses carrying shRNA were produced and 786-O cells were infected as described above. Stable cell pools were obtained after selection with 1 μg/mL puromycin (Beyotime Biotechnology, ST551). The shRNA sequences used for targeting human *AXL* were listed below:

shAXL-3: 5’-CGAAAGAAGGAGACCCGTTAT-3’

shAXL-5: 5’-GCTGTGAAGACGATGAAGATT-3’

### High-throughput screening of ferroptosis inhibitors

A high-throughput screening was performed as described [2]. In brief, a total of 500,000 HT-1080 cells were seeded into 96-well plates and treated with erastin or DMSO. An automatic sample injector was applied to add testing compounds into cells, and every well was treated with an individual compound. After 24 h, viabilities of cells in 96-well plates were assayed. The iron chelator DFO was used as a positive control. The corresponding compounds that showed comparable viabilities to DFO treatment were decoded for further analysis.

### Cell viability assay

Cell viability was evaluated by measuring the cellular ATP level using a CellTiter-Lumi^TM^ Plus Luminescent Cell Viability Assay Kit (Beyotime Biotechnology, C0068) according to the manufacturer’s instruction. Cell viability is reported as a percentage relative to the negative control treatment. The curves were fitted using a nonlinear regression model.

### Cell death assay

Cell death was evaluated by PI staining and FACS analysis. PI was added at a final concentration of 2 μg/mL into medium at 1 h before sample collection. The cells in 12-well plate were then digested by trypsin and centrifuged. The cell pellet was suspended with 2% Paraformaldehyde (in PBS). The PI signal of cells was detected in PE channel.

### Glutathione assay

The concentrations of total intracellular GSH and GSSG were detected using the Total Glutathione Assay Kit (Beyotime Biotechnology, S0052) following the manufacturer’s instruction. The total glutathione content of samples at the time of determination can be calculated by comparing the standard curve.

### Total antioxidant capacity assay

The antioxidant capacity of indicated compounds were detected using Total Antioxidant Capacity Assay Kit with ABTS method (Beyotime Biotechnology, S0121) following the manufacturer’s instruction. Trolox was used as a positive reference for the total antioxidant capacity of other antioxidants. Every indicating compound used in this ABTS test was 1 mM. The total antioxidant capacity was calculated according to the standard curve made by Trolox.

### DPPH assay

2,2-Di(4-tert-octylphenyl)-1-picrylhydrazyl (DPPH) was used to measure the radical scavenging activities of chemicals, based on the color changes from deep purple to pale yellow when it reacts with antioxidant molecules. Briefly, DPPH was dissolved in methanol to a final working concentration of 0.05 mM. 1 ml of DPPH solution was added to each test compound dissolved in DMSO, whose final concentration was 0.05 mM. Samples were then mixed well and kept in dark place at room temperature for 30 min. Then determined absorbance at A517 with a microplate reader.

### Cellular lipid-ROS assay

Cellular lipid-ROS was measured using fluorescent dye C11-BODIPY 581/591. Cells were seeded in 12-well cell plates followed the indicated treatments. After treatment, C11-BODIPY 581/591 was added to the culture medium at a final concentration of 5 μM and cell samples were incubated at 37 °C for 30 min in the dark. Subsequently, cells were harvested and fixed in PBS containing 2% paraformaldehyde. After strained through a 40-μm cell strainer, cells were analyzed by flow cytometer (Accuri C6, BD Biosciences) equipped with 488 nm laser for excitation. Data were collected from the FITC channel. A minimum of 10,000 cells were analyzed per condition.

### Autoxidation of eggPC liposomes assay

EggPC (egg phosphatidyl-choline) lipids liposomes (1 mM) and C11-BODIPY 581/591 (1 µM) were added to a black 96-well polypropylene plate in PBS at pH 7.4. This was followed by the addition of either Lip-1 (1 µM), (R)-CRZ (1 µM), or (S)-CRZ (1 µM) to the reaction individually. The plate was incubated for 5 min and initiated by adding 1 mM AAPH. Fluorescence signal of C11-BODIPY was monitored by excitation at 488 nm and emission at 518 nm using microplate reader (PerkinElmer).

### Cellular ferrous ion level detection

Cells (1.5 × 10^6^ cells/plate) were seeded on a black 96-well-plate and cultured overnight. Cells were treated with (S)-CRZ (3 μM) or (R)-CRZ (3 μM) or DFO (100 μM) for 7 h, and washed 3 times with PBS. Then, serum-free DMEM (50 µl) containing 1 µM FerroOrange (DOJINDO, F374) was added to the cells. After incubation at 37 °C for 30 min, the fluorescent signal was measured by microplate reader (Ex = 543 nm, Em = 580 nm).

### Kidney ischemic-reperfusion injury mouse model

C57BL/6 J male mice aged 8 weeks were purchased from Charles River Laboratories International, Inc., and housed in a specific pathogen-free animal facility. Mice were randomly intraperitoneal preinjected with DMSO or Fer-1 (5 mg/kg), (R)-CRZ (4 mg/kg), (R)-CRZ (10 mg/kg) or (S)-CRZ (4 mg/kg) for 2 h, followed by induction of kidney ischemic-reperfusion injury which was performed via a midline abdominal incision and bilateral renal pedicles clamping for 40 min. The body temperature of mice was maintained around 37 °C during the entire surgical procedure. Mice were sacrificed 24 h after operation, right kidney and serum were collected at this time point for hematoxylin and eosin (H & E) staining, IHC staining and measurement of BUN and Creatinine.

For the scoring system, tissues were stained with H & E, and the degree of morphological involvement in renal failure was determined using light microscopy. The following parameters were chosen as indicative of morphological damage to the kidney after ischemia-reperfusion injury (IRI): brush border loss, tubule dilatation, tubule degeneration, tubule necrosis, and tubular cast formation. These parameters were evaluated on a scale of 0-10, which ranged from not present (0), mild (0–3.5), moderate (3.5–6.5), and severe (6.5–10).

For IHC staining, tissue sections were deparaffinized, hydrated, and subjected to antigen retrieval using citrate antigen retrieval solution (Beyotime Biotechnology, P0083). After blocking with 5% goat serum at room temperature for 1 h, the sections were incubated overnight with a 4-HNE antibody (Abcam, ab46545, 1:100), followed by incubation with a secondary antibody. Subsequently, DAB substrate was applied until the desired stain intensity developed. The sections were counterstained with hematoxylin, differentiated, visualized and mounted in synthetic resins.

### Lipidomic analysis in HT-1080 cells

HT-1080 cells cultured in plates were treated with (S)-CRZ (3 µM) or Vehicle for 72 h, 3 × 10^6^ cells were collected as one sample. Each group has 4 independent repeat samples for testing. Lipidomic analysis was carried out by the LipidALL Technologies Co., Ltd. (Changzhou, China).

### Proliferation of cultured B16-F10 cells

B16-F10 cells were attached to the plate after 24 h of culture. The corresponding drugs were added to a working concentration of 1 µM. Changed fresh medium every 24 h and added corresponding drugs to its working concentration. Numbers of viable cells were count by Trypan Blue staining. Cell counts were calculated at 0 h, 48 h and 96 h after cells were seeded.

### B16-F10 subcutaneous xenograft mice model

C57BL/6 J male mice aged 6 weeks were injected subcutaneously on the right back flank with 5×10^5^ B16-F10 cells. Second day after tumor implantation, mice were random allocated into 4 groups (5/group) and intraperitoneally injected with indicated drugs (Lip-1:10 mg/kg/day, (S)-CRZ & (R)-CRZ:5 mg/kg/day, control group was treated with vehicle solution). Since each compound has low solubleness in water, all of them were dissolved in DMSO, and used a protocol of (10% DMSO + 30% PEG300 + 5% Tween 80 + 55% PBS) as vehicle to delivered daily drugs. The tumor growth was monitored from the Day 6 till the end point.

At the end point, mice were scarified with euthanasia. Tumors, TdLNs and nTdLNs were excised from mice. Lymph cells were collected by grinding lymph nodes in PBS containing 2% FBS followed by staining for flow cytometry analysis.

For ex vivo assay, B16-F10 cells were implanted in 8 male C57BL/6 J mice in the same method as in vivo assay experiment described. Monitored tumor growth till the end point. Collected lymph cells from TdLNs and nTdLNs respectively. Mixed lymph cells from TdLNs well and counted alive cells with trypan blue staining. Each sample contained 1×10^6^ lymph cells and treated with DMSO, Lip-1 (2 µM), (S)-CRZ (2 µM) or (R)-CRZ (2 µM) at room temperature for 3 h followed by staining for flow cytometry analysis. Each group has 3 independent repeats. The same treatment also performed in lymph cells from nTdLNs.

### Flow cytometry

Stained the lymph cells with CD45, CD3e, CD8 and CD4 antibodies, as well as probes including Fixable Viability Stain 510 and C11-BODIPY 581/591. Surface staining was conducted according to manufacturer’s instructions. The accumulation of lipid-ROS in subgroups of T cells was detected in FITC channel.

### Statistical analysis

Results were analyzed using GraphPad Prism 10.0, CytExpert, and FlowJo. The fluorescence signals of flow cytometry were measured using CytExpert and FlowJo. Statistical analyses were performed using one-way analysis of variance (ANOVA) with Dunnett’s multiple comparisons test (Figs. 2A, B, F–H, 3B, C, E, F, 5A, B, D, G, 6B, G, Supplemental Fig. 1A, B, Supplemental Fig. 3B, C) or Holm-Šídák’s multiple comparisons test (Fig. 6F) or Tukey’s multiple comparisons test (Fig. 1D). Other statists were analyzed using two-way ANOVA with Dunnett’s multiple comparisons test (Figs. 1B, E, G, J, 2E, 6A, C, D, Supplemental Fig. 1C, Supplemental Fig. 3A). All data shown represent the results obtained from at least triplicated independent experiments with standard errors of the mean (mean ± SD), ****p < 0.0001, *** *p* < 0.0002, ** *p* < 0.0021, * *p* < 0.0332. Statistical analyses of IC_50_ were calculated by the nonlinear regression analysis with the dose-response-inhibition models and chose: (log(inhibition) vs. response-variable slope (four parameters)) to draw fitted curves. Statistical analyses of EC_50_ were calculated by the nonlinear regression analysis with the dose-response-stimulation models and chose: (log(agonist) vs. response-variable slope (four parameters)) to draw fitted curves.

### Supplementary information

Supplementary information

## Data Availability

All data are available in the main text or the supplementary materials. Supplemental Figs. S1–3 (PDF): Viability and death of cells with indicated treatment; Gene sequencing result of *AGPAT3* knockout monoclonal cell lines and cell sensitivity to ferroptosis with either AGPAT3 overexpression or knockout; Cellular lipid-ROS in CD4^+^ T cells from TdLN and nTdLN of tumor-bearing mice. Uncropped original western blots.
