# Supplementary material for: Crizotinib and its enantiomer suppress ferroptosis by decreasing PE-O-PUFA content
Source: Cell Death Discov. 2024 Aug 12;10:360. doi: 10.1038/s41420-024-02127-8 (PMC11319649; doi:10.1038/s41420-024-02127-8)
Supplement: Supplementary file 1 — Supplementary information [file 41420_2024_2127_MOESM1_ESM.pdf]

Supporting information

Crizotinib and its enantiomer suppress ferroptosis by decreasing PE-O-PUFA content

Si-Yu Cen[1], Fang Lin[2,3], Xuan Li[2,3], Yanglin Hu[4], Jin-Pin Liu[1], Zi-An Xue[5], Yun Gao[1], Yi-Ping Sun[1], Sanyong Zhu[3], Yongjun Dang[3], Yahui Zhao[2,3] and Hai-Xin Yuan[1,2,3]*

[1] The Fifth People's Hospital of Shanghai, Molecular and Cell Biology Laboratory, Institutes of Biomedical Sciences, Fudan University, Shanghai 200032, China

[2] College of Pharmacy & Department of Cancer Center, the Second Affiliated Hospital, Chongqing Medical University, Chongqing 400016, China

[3] Basic Medicine Research and Innovation Center for Novel Target and Therapeutic Intervention (Ministry of Education), Chongqing Medical University, Chongqing 400016, China

[4] Department of nephrology, Wuhan No.1 hospital, Wuhan 430022, China

[5] Department of Medicinal Chemistry, School of Pharmacy, Fudan University, Shanghai 200120, China

*Correspondence to: Hai-Xin Yuan (yuanhaixin@fudan.edu.cn)

Supporting information

Supplemental Figures S1-S3: Viability and death of cells with indicated treatment; Gene sequencing result of *AGPAT3* knockout monoclonal cell lines and cell sensitivity to ferroptosis with either AGPAT3 overexpression or knockout; Cellular lipid-ROS in $CD4^+$ T cells from TdLN and nTdLN of tumor-bearing mice.

Uncropped original western blots.

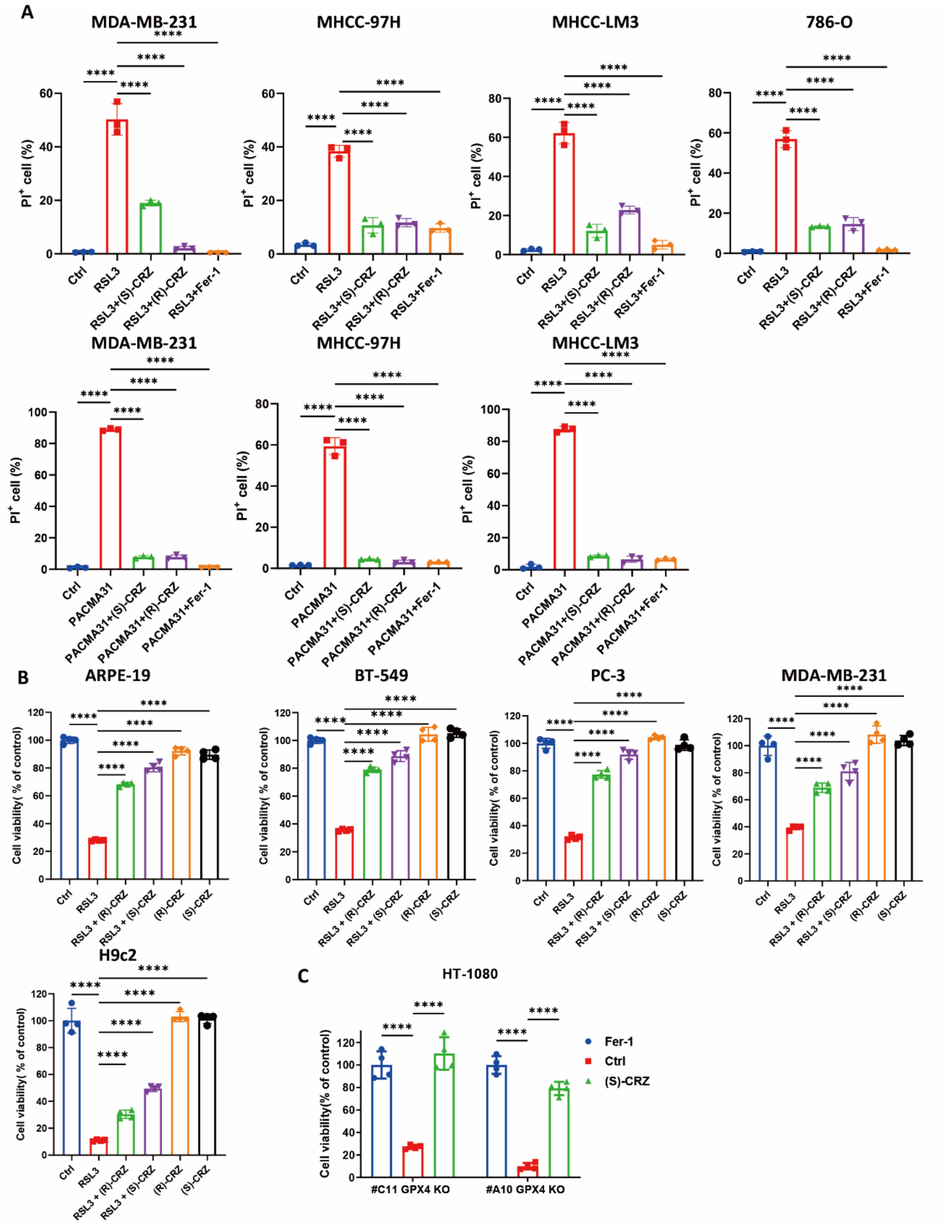

Supplemental Figure 1. Enantiomers of CRZ protect cells from ferroptosis

(A-B) (S)-CRZ (1 μM) and (R)-CRZ (1 μM) inhibit ferroptosis in multiple cell lines. Cell death (A) and cell viability (B) were detected at appropriate time. ARPE-19, human retinal

pigmented epithelium; BT-549, epithelial cells from invasive breast ductal tumor; MDA-MB-231, epithelial-like cell of breast adenocarcinoma; PC-3, prostatic adenocarcinoma. (C) (S)-CRZ (3 μM) inhibits spontaneous ferroptosis in *GPX4* KO monoclonal HT-1080 cell lines. Cell viability was measured after 12 h of treatments. Data in Supplemental Figure 1 were presented as the mean ± S.D., n = 3 independent repeats in Supplemental 1A, n = 4 independent repeats in Supplemental Figure 1B and 1C.

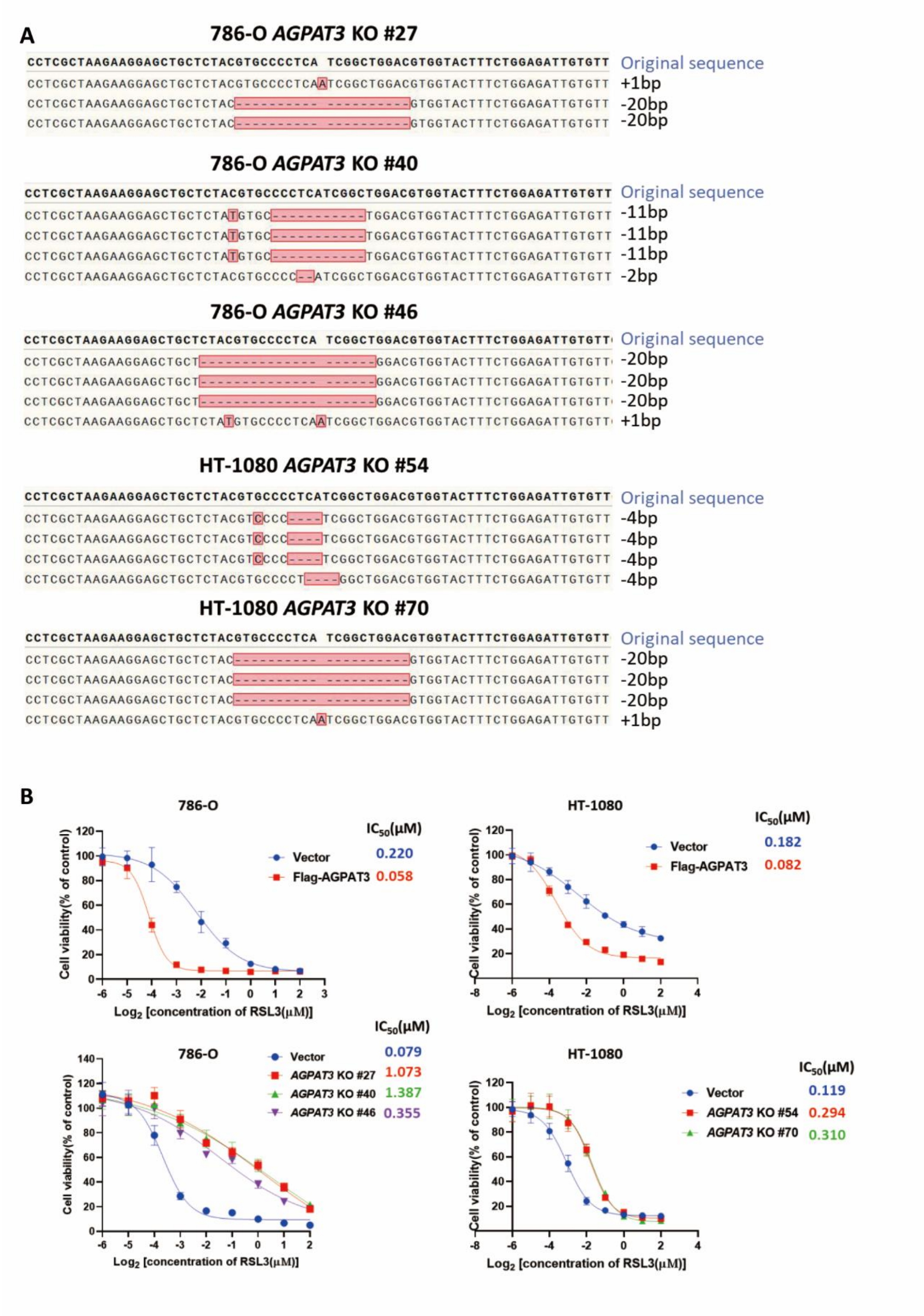

Supplemental Figure 2. ***AGPAT3*** **gene knockout increase the resistance to ferroptosis**

(A) Sanger gene sequencing technology was used to identify the knockout effect of the *AGPAT3* gene in various monoclonal cell strains. (B) AGPAT3 overexpression increased

cell sensitivity to ferroptosis, while *AGPAT3* knockout rendered cells more resistant to ferroptosis. Cell viability of HT-1080 was measured after 8 h of treatments. Cell viability of 786-O was measured after 10 h of treatments. Data in Supplemental Figure 2B were presented as the mean ± S.D., n = 3 independent repeats.

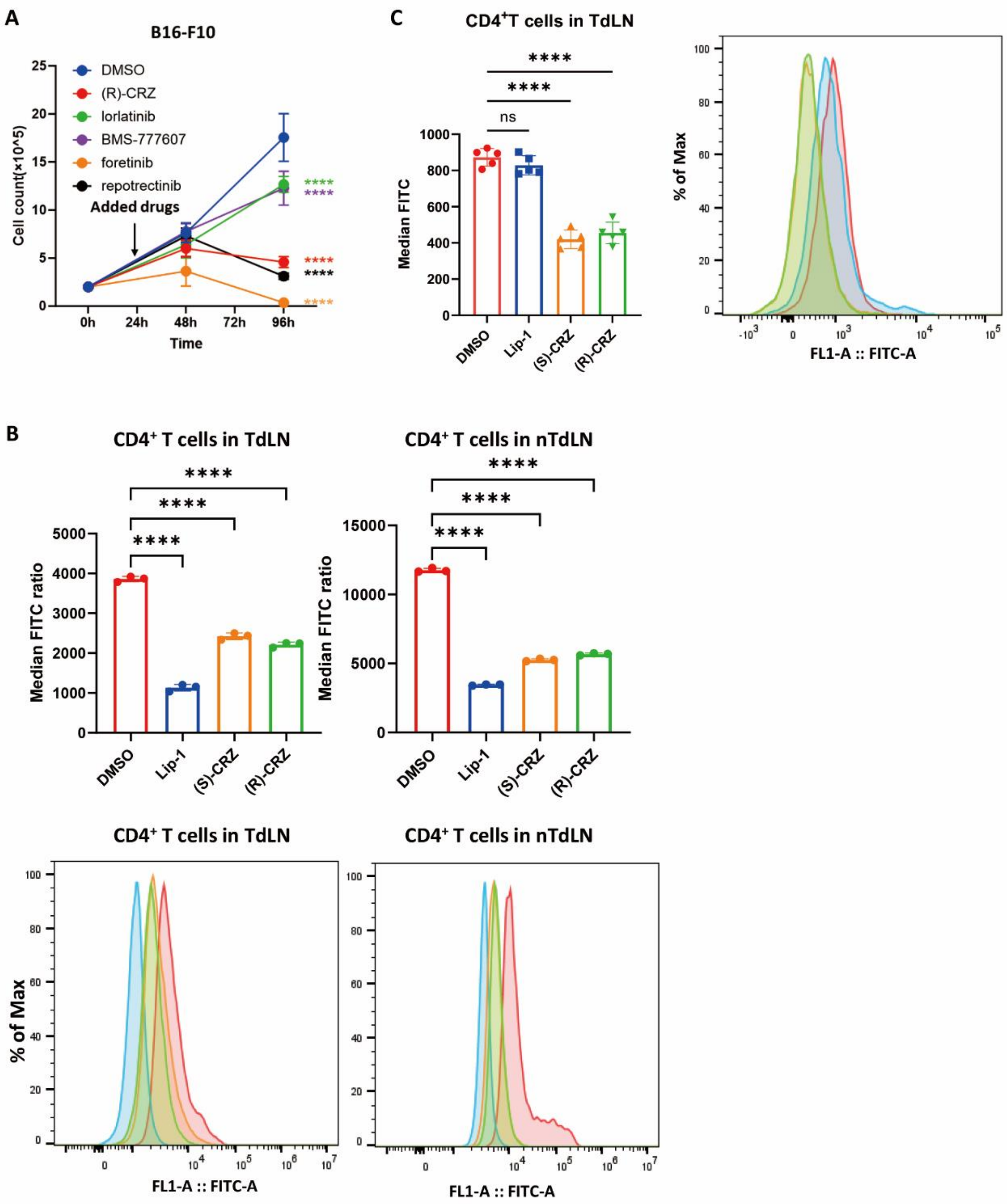

Supplemental Figure 3. (S)-CRZ and (R)-CRZ eliminate lipid-ROS in CD4$^+$ T cell

(A) The proliferation curves of B16-F10 cells with different drug treatments. Groups were distinguished by color, and statistical significance was assessed by comparing differences with the DMSO group as a control at 96 h. (B) Lip-1 (2 µM), (S)-CRZ (2 µM) and (R)-CRZ (2 µM) treatments reduce lipid-ROS in CD4$^+$ T cells isolated from TdLNs and nTdLNs of B16-F10 tumor subcutaneous bearing mice. Lymph cells from lymph nodes

separated from tumor-bearing mice were treated with the indicated compounds for 3 h followed by flow cytometry analysis. Lipid-ROS was detected by fluorescence of C11-BODIPY 581/591 at the FITC channel. Data in Figures S3A-S3B were presented as the mean ± S.D., n = 3 independent repeats. (C) (S)-CRZ (5 mg/kg) and (R)-CRZ (5 mg/kg) treatment significantly reduce lipid-ROS in $CD4^+$ T cells from TdLNs of B16-F10 tumor subcutaneous bearing mice. $CD4^+$ T cells were isolated from TdLNs of tumor-bearing mice with intraperitoneal injection of indicated drug for 16 d. Lipid-ROS was detected by fluorescence of C11-BODIPY 581/591 at the FITC channel. Data was presented as the mean ± S.D., n = 5 independent repeats.

Figure 1F

Original Western blot

IB: ALK

IB: β-actin

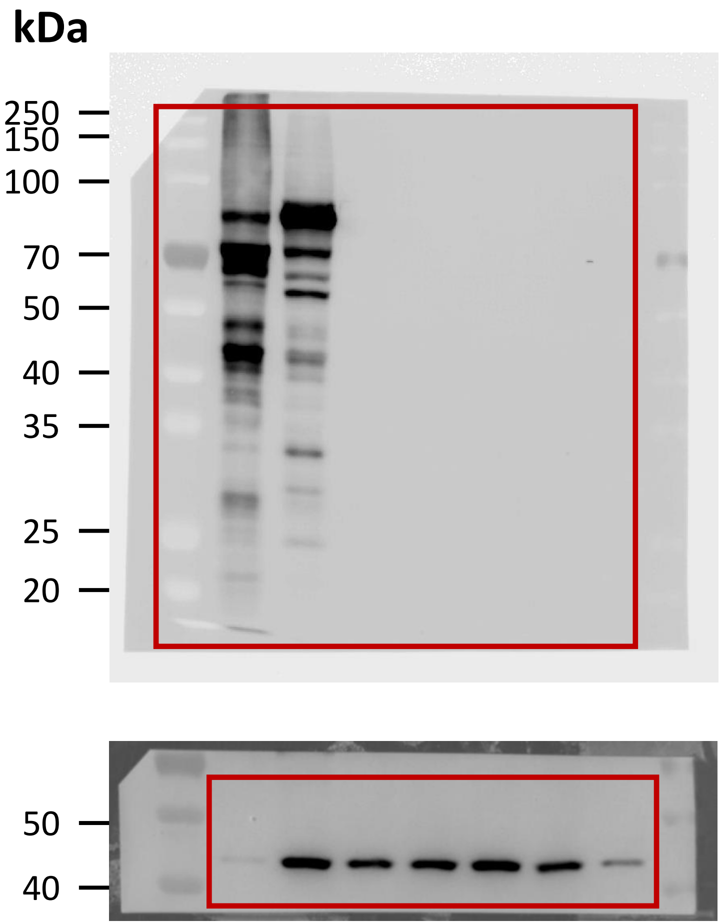

Figure presented in manuscript

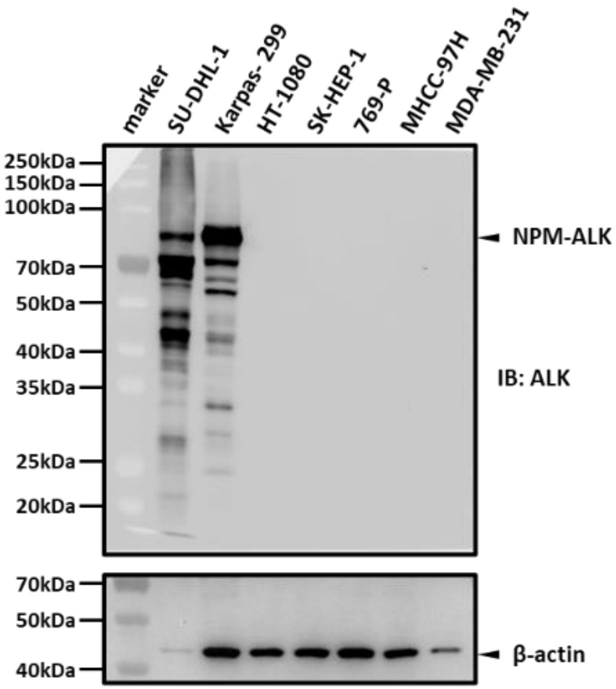

Colorimetric and chemiluminescent were merged.

Red boxes indicate the cropped areas that were used in the figure.

Figure 1H

Original Western blot

Figure presented in manuscript

kDa

IB: AXL

IB: β-actin

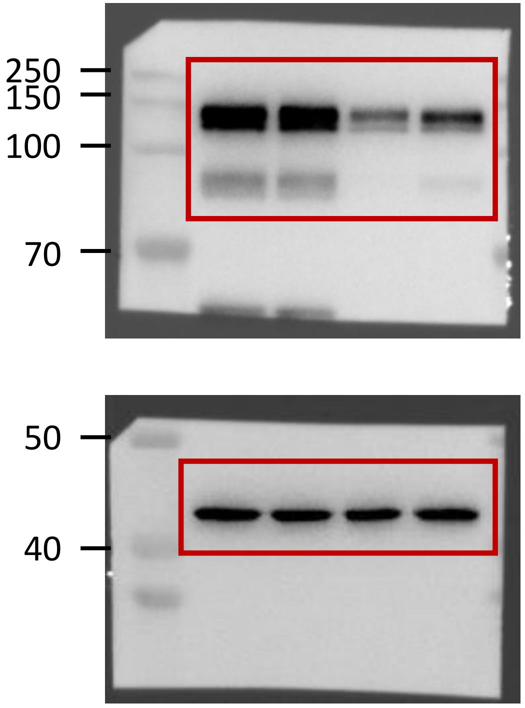

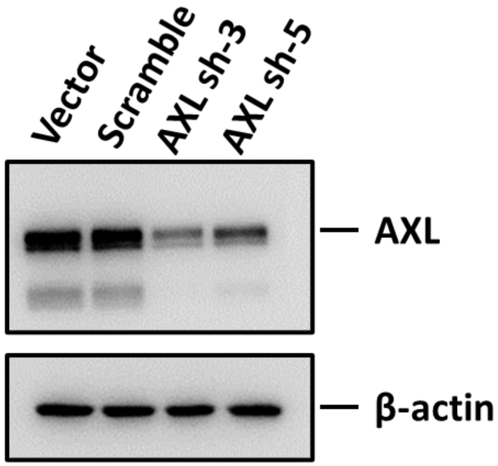

Membrane used for β-actin detection was cut from the same membrane for AXL.
Colorimetric and chemiluminescent were merged.
Red boxes indicate the cropped areas that were used in the figure.

Figure 1I

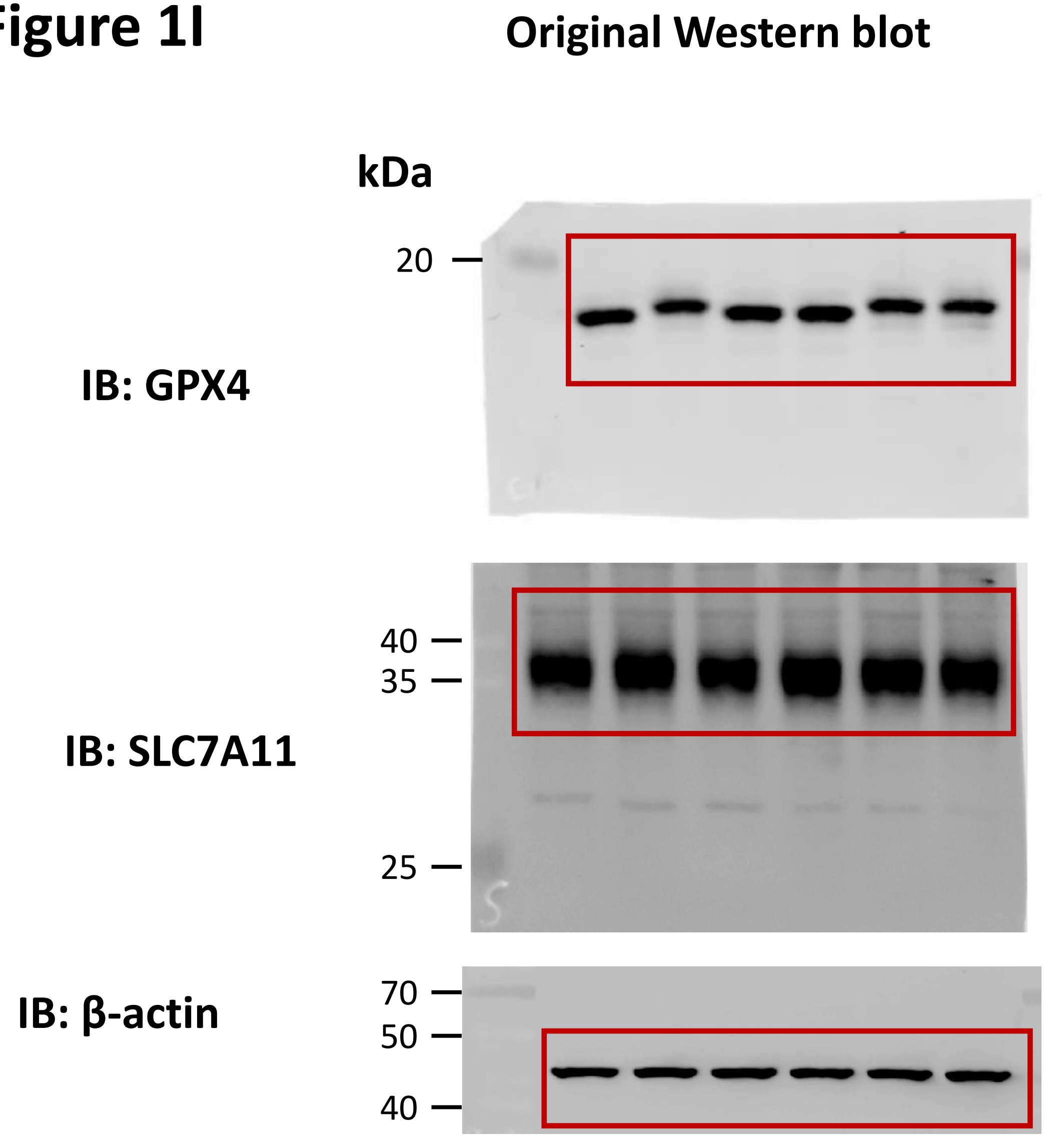

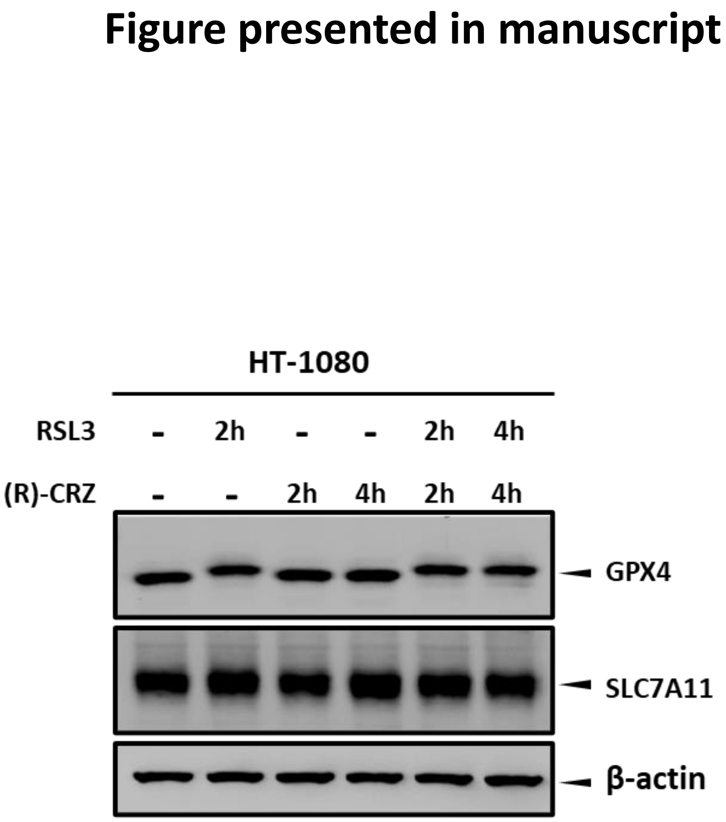

Colorimetric and chemiluminescent were merged.

Red boxes indicate the cropped areas that were used in the figure.

Figure 1J

Original Western blot

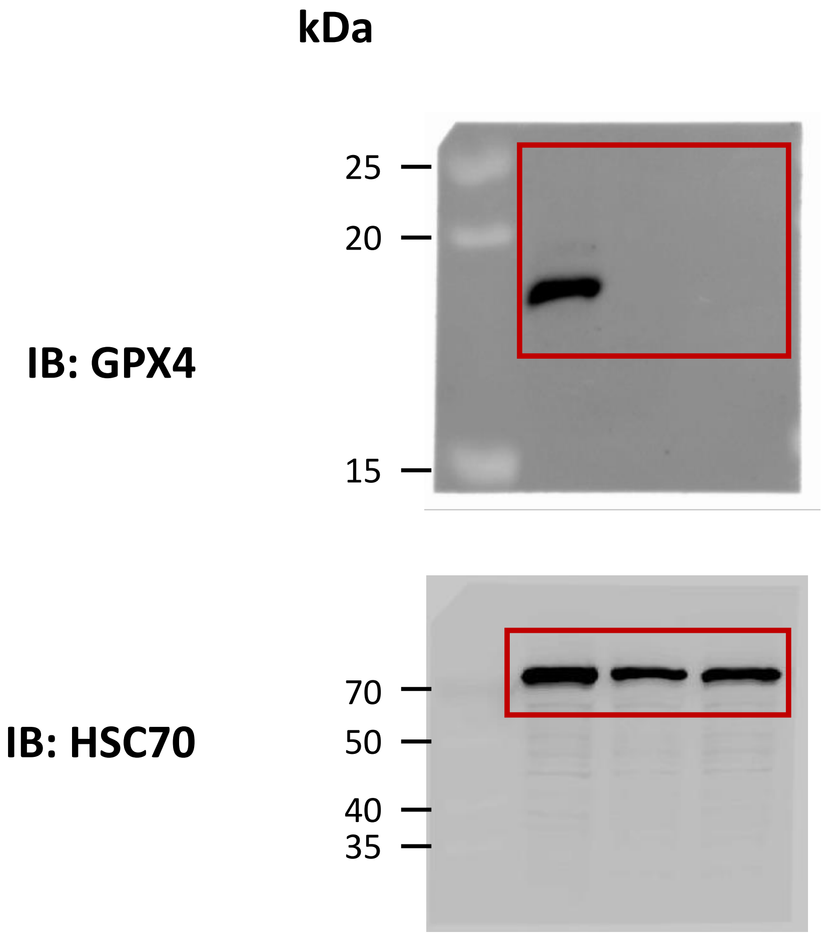

Figure presented in manuscript

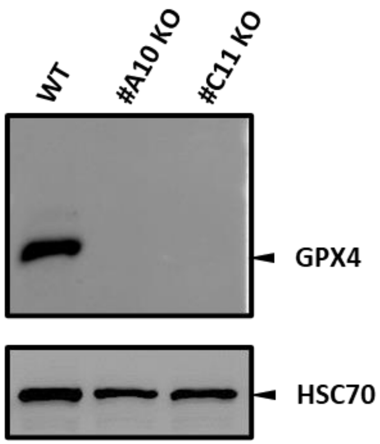

Colorimetric and chemiluminescent were merged.

Red boxes indicate the cropped areas that were used in the figure.

Figure 3G

Original Western blot

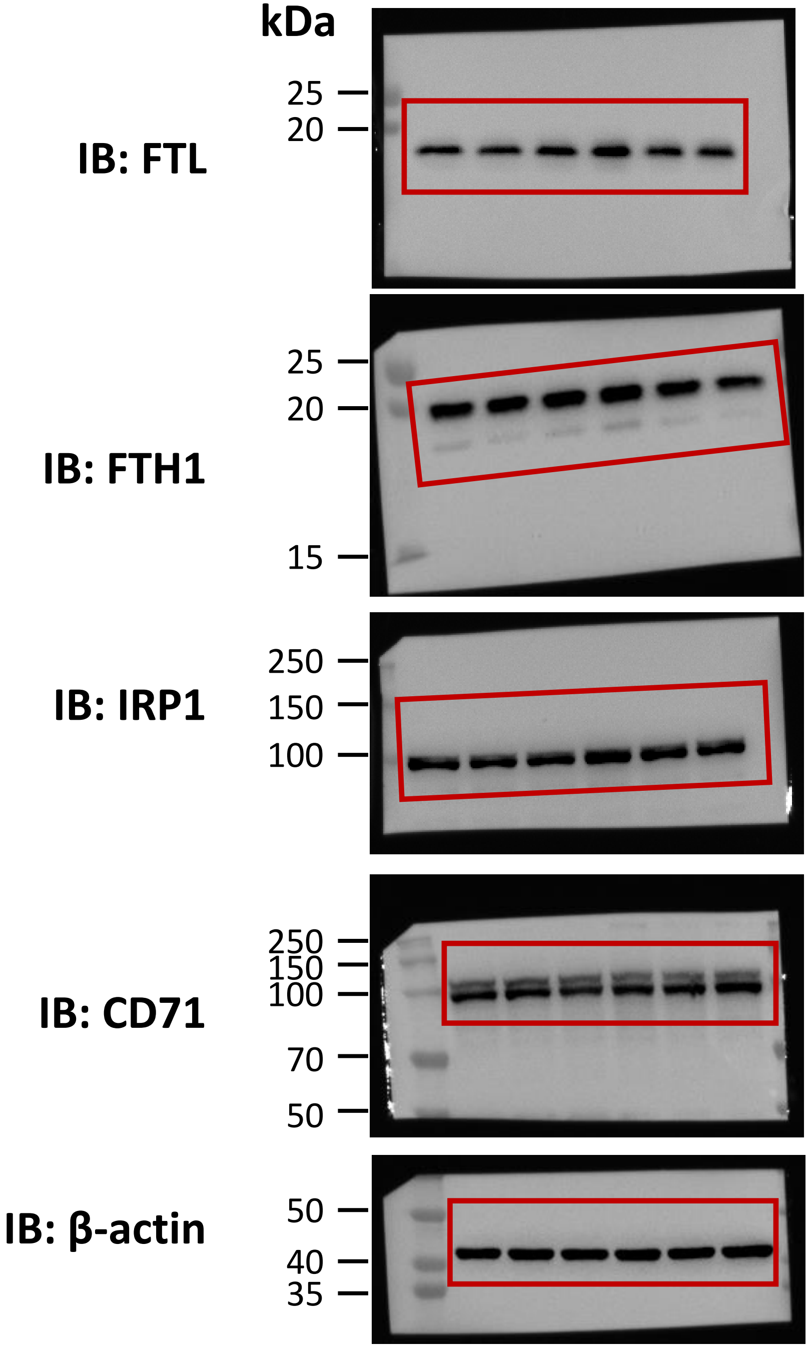

Figure presented in manuscript

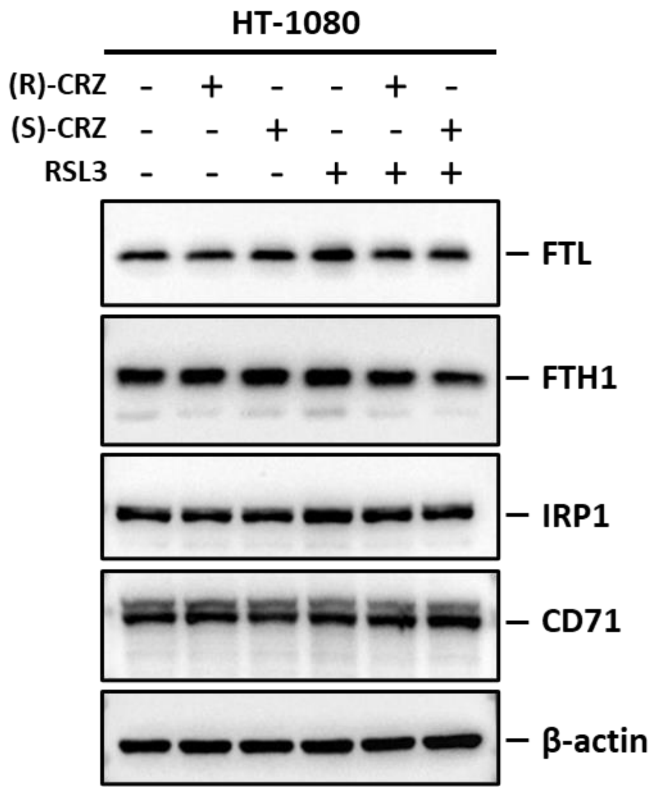

Colorimetric and chemiluminescent were merged.

Red boxes indicate the cropped areas that were used in the figure.

Figure 4A

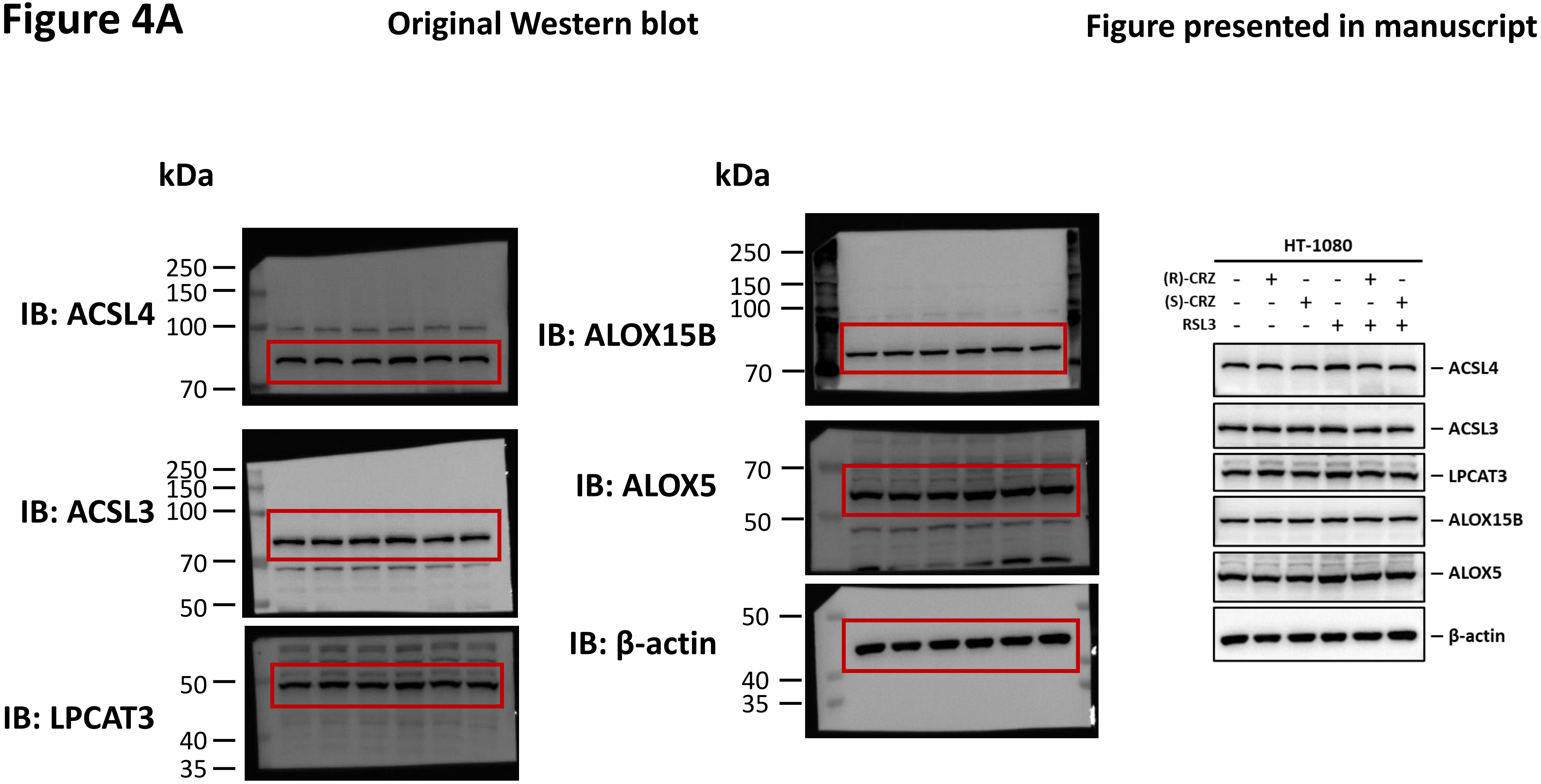

Colorimetric and chemiluminescent were merged.

Red boxes indicate the cropped areas that were used in the figure.

Figure 4F

Original Western blot

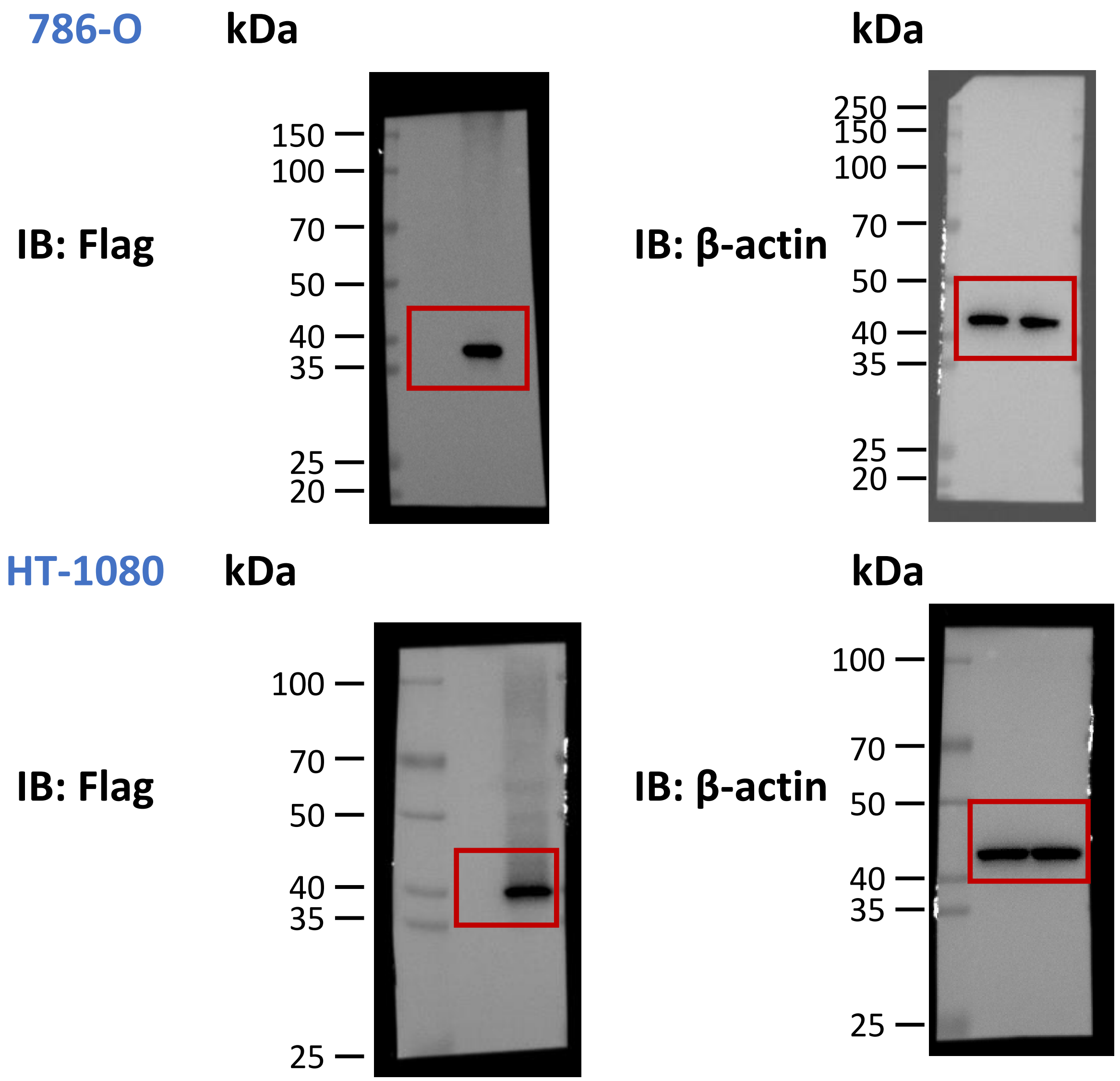

Figure presented in manuscript

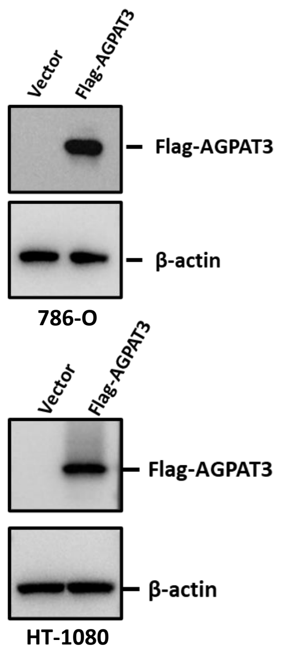

Colorimetric and chemiluminescent were merged.

Red boxes indicate the cropped areas that were used in the figure.
